# Heterogeneous miRNA-mRNA Regulatory Networks of Visceral and Subcutaneous Adipose Tissue in the Relationship Between Obesity and Renal Clear Cell Carcinoma

**DOI:** 10.3389/fendo.2021.713357

**Published:** 2021-09-21

**Authors:** Yuyan Liu, Yang Liu, Jiajin Hu, Zhenwei He, Lei Liu, Yanan Ma, Deliang Wen

**Affiliations:** ^1^Institute of Health Sciences, China Medical University, Shenyang, China; ^2^Department of Clinical Epidemiology, The Fourth Affiliated Hospital of China Medical University, Shenyang, China; ^3^Department of Biostatistics and Epidemiology, School of Public Health, China Medical University, Shenyang, China

**Keywords:** renal clear cell carcinoma, obesity, adipose tissue, micro RNA, messenger RNA

## Abstract

**Background:**

Clear cell renal cell carcinoma (ccRCC) is one of the most lethal urologic cancer. Associations of both visceral adipose tissue (VAT) and subcutaneous adipose tissue (SAT) with ccRCC have been reported, and underlying mechanisms of VAT perhaps distinguished from SAT, considering their different structures and functions. We performed this study to disclose different miRNA-mRNA networks of obesity-related ccRCC in VAT and SAT using datasets from Gene Expression Omnibus (GEO) and The Cancer Genome Atlas (TCGA); and find out different RNAs correlated with the prognosis of ccRCC in VAT and SAT.

**Methods:**

We screened out different expressed (DE) mRNAs and miRNAs of obesity, in both VAT and SAT from GEO datasets, and constructed miRNA-mRNA networks of obesity-related ccRCC. To evaluate the sensitivity and specificity of RNAs in networks of obesity-related ccRCC in both VAT and SAT, Receiver Operating Characteristic (ROC) analyses were conducted using TCGA datasets. Spearman correlation analyses were then performed to find out RNA pairs with inverse correlations. We also performed Cox regression analyses to estimate the association of all DE RNAs of obesity with the overall survival.

**Results:**

136 and 185 DE mRNAs of obesity in VAT and SAT were found out. Combined with selected DE miRNAs, miRNA-mRNA networks of obesity-related ccRCC were constructed. By performing ROC analyses, RNAs with same trend as shown in networks and statistically significant ORs were selected to be paired. Three pairs were finally remained in Spearman correlation analyses, including hsa-miR-182&ATP2B2, hsa-miR-532&CDH2 in VAT, and hsa-miR-425&TFAP2B in SAT. Multivariable Cox regression analyses showed that several RNAs with statistically significant adjusted HRs remained consistent trends as shown in DE analyses of obesity. Risk score analyses using selected RNAs showed that the overall survival time of patients in the low‐risk group was significantly longer than that in the high‐risk group regardless of risk score models.

**Conclusions:**

We found out different miRNA-mRNA regulatory networks of obesity-related ccRCC for both VAT and SAT; and several DE RNAs of obesity-related ccRCC were found to remain consistent performance in terms of ccRCC prognosis. Our findings could provide valuable evidence on the targeted therapy of obesity-related ccRCC.

## Introduction

Renal cell carcinoma (RCC) is one of the most lethal urologic cancer (1), encompassing histological subtypes of cancers derived from renal tubular epithelial cells (2). Approximately 2% of incidence and mortality of malignances can be attributed to RCC (3). Clear cell renal cell carcinoma (ccRCC) is the most common subtype of RCC, accounting for over 70% of cases (4). Obesity has been identified as one of the independent modifiable risk factors of RCC, and is involved in the development of more than 30%-40% of RCC cases (5). Published studies demonstrated that ccRCC risk was 1.5-fold higher in people with obesity than those with normal weight (6). Another longitudinal cohort study found that after an average follow-up of 11.3 years, large body mass index (BMI) at baseline was associated with the increased risk of ccRCC, hazard ratio (HR) per 1 kg/m^2^ increase of BMI was 1.09 with the 95% confidence interval (CI) of (1.02, 1.16) (7). Inflammatory cytokines released from adipose tissue has been considered playing a key role in the pathophysiology of obesity-related ccRCC (6). Results from a case-control study including 682 ccRCC cases implied that insulin resistance and inflammation driven by several obesity-related biomarkers might underlie the relationship between obesity and ccRCC (8).

Abdominal adipose tissue, including visceral adipose tissue (VAT) and subcutaneous adipose tissue (SAT), is not only a main storage of fat in mammals, but also an important endocrine organ secreting adipokines, such as cytokines, interleukins and other biologically active molecules (9). Both VAT and SAT have been reported to be positively associated with the prevalence/incidence of several cardiovascular diseases (10) and cancers (11, 12), including ccRCC. Greco, et al. found that both VAT and SAT areas measured using computed tomography (CT) were larger in ccRCC group compared to controls (13). However, no study has revealed if the mechanism of VAT underlying the relationship between obesity and ccRCC distinguished from that of SAT. We thought such heterogeneity perhaps existed, considering differences in levels of lipid mobilization, adipokine production and adipocyte differentiation between VAT and SAT (14, 15).

It has been well proven that gene expression profiles estimated using message RNA (mRNA) level could be an effective method to predict the pathophysiological mechanisms of several cancers (16–18). For an instance, a bioinformatic analysis performed by Zhou et al., showed that 5 genes, such as IGHA1 and IGKC were correlated with ccRCC (19). In addition, micro RNA (miRNA) is a class of well-characterized RNA without protein encoding potential. miRNAs can bind to their target genes, typically resulting in gene silencing by triggering the degradation of the target mRNAs, and associations of miRNAs with ccRCC have been reported (20–22). However, no miRNA-mRNA regulatory network relative to obesity-related ccRCC was given. On the other hand, whether ccRCC-relevant RNAs were different between VAT and SAT remained unknown.

**Figure 1:**
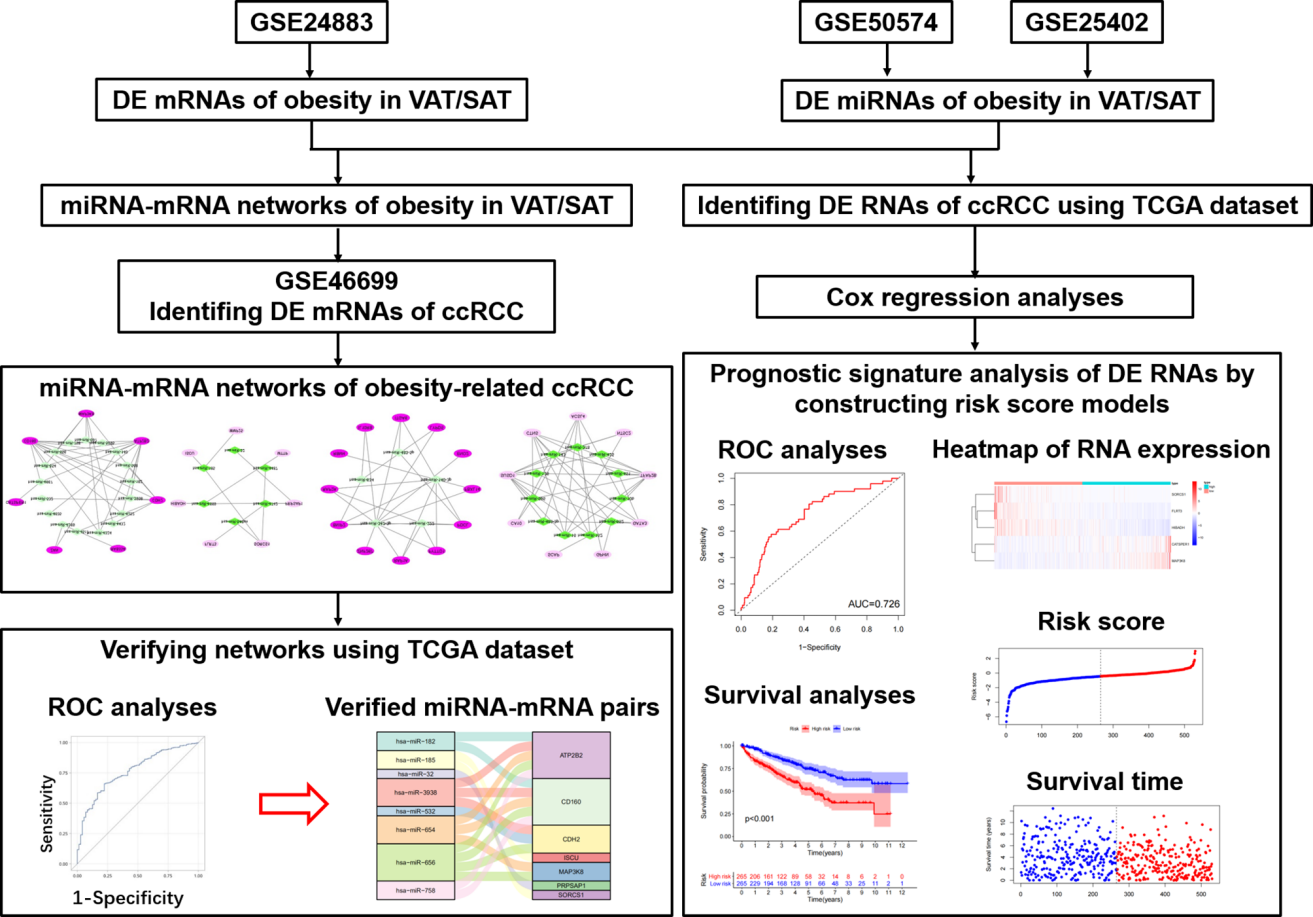
Flow chart of this study.

Therefore, as shown in **Figure 1**, we performed this study aiming to disclose different miRNA-mRNA networks of obesity-related ccRCC in VAT and SAT using datasets from Gene Expression Omnibus (GEO); verify the obtained miRNA-mRNA pairs relevant to ccRCC using the dataset of The Cancer Genome Atlas (TCGA); and find out different mRNAs and miRNAs correlated with the prognosis of ccRCC in VAT and SAT. Findings in this study would be helpful to distinguish the molecular mechanisms of obesity-related ccRCC between VAT and SAT, and provide valuable evidence on the targeted therapy of obesity-related ccRCC.

## Methods

### Selecting Microarray Datasets and Collecting Data

We first downloaded the microarray dataset (GSE24883) of mRNA expression profiles in both VAT and SAT based on the platform of GPL4133 from the GEO (https://www.ncbi.nlm.nih.gov/geo/) (23). The GSE24883 dataset included 64 samples of VAT, as well as paired SAT from 32 female participants, who were divided in 4 subgroups according to BMI, and the presence of the metabolic syndrome (MS) (24), i.e. lean (8/32), overweight (8/32), obesity (8/32), and obesity with MS (8/32). Whole transcriptome analysis was performed using DNA microarrays on VAT and SAT samples, and total RNA was hybridized to Agilent 44k whole human genome microarray (Agilent, Santa Clara, CA). Thereafter, miRNA expression profiles of VAT and SAT were obtained from datasets of GSE50574 (GPL16384) and GSE25402 (GPL8786), respectively. In GSE50574 datasets, 7 obese (BMI: 33-50 kg/m^2^) and 5 lean (BMI: 22-25 kg/m^2^) samples were included. Adipocyte-exosomal total RNA was extracted from VAT, and only mature human miRNAs were retained as Affymetrix 3.0 miRNA arrays (Affymetrix, Santa Clara, CA) (25). GSE25402 dataset included 30 obese (BMI> 30 kg/m^2^) and 26 non-obese samples (BMI< 30 kg/m^2^) healthy samples. Total RNA was extracted from abdominal subcutaneous white adipose tissue, and miRNAs were retained as Affymetrix multispecies miRNA-1 array (Affymetrix) (26). In addition, mRNA expression profiles of ccRCC (GSE46699, GPL570) was downloaded, and only 24 samples (patient-matched tumors and adjacent-normal tissues) from 12 obese participants (BMI> 30 kg/m^2^) were used for the following analyses, considering that we would like to find out if differentially expressed (DE) mRNAs of obesity in both VAT and SAT datasets were correlated with the presence of obesity-related ccRCC (27).

### Identifying DE mRNAs and miRNAs of Obesity in VAT and SAT

Firstly, we downloaded the series matrix file of GSE24883 and the platform file of GPL4133. In accordance with the annotations, probe IDs in the matrix file were transformed into the gene symbols, and probes that did not correspond to the mRNA symbols were excluded. The average value of mRNA correlated with more than one probe was used as the final expression value (28). We then normalized the data of mRNA expression profiles using robust multi-array average (RMA) based on the limma package in R (v.4.0.3) software (29). Since samples in GSE24883 were divided into 4 subgroups, statistical comparisons were respectively performed between lean and other 3 subgroups with different obese statuses using the Bayesian method in limma package. By setting a cutoff threshold of P-value < 0.05 and |log_2_fold change (FC)| > 2, DE mRNAs of overweight, obesity and obesity with metabolic syndrome (MS) in both VAT and SAT were revealed. Consistent methods as above mentioned were respectively repeated in both GSE50574 and GSE25402 datasets to obtain the DE miRNA of obesity in VAT and SAT using the cutoff of P value < 0.05 and |FC| > 1.2 (25). Relevant volcano plots and heatmaps of both mRNAs and miRNAs were given using R software. Furthermore, for DE mRNAs of 3 obese statuses, Venn diagrams were generated using VennDiagram R package to disclose the common DE mRNAs in 3 obese statuses in both VAT and SAT.

### Constructing miRNA-mRNA Networks of Obesity in VAT and SAT

Based on the up- and down-regulated DE miRNAs of obesity in VAT and SAT, miRNA-mRNA pairs were predicted using Targetscan database (30), followed by performing VennDiagram R package to obtain the intersections between predicted mRNAs in miRNA-mRNA pairs and DE ones of obesity in VAT and SAT, respectively. Secondly, we screened out the pairs including the DE mRNAs in the intersection, and then only retained those showing inverse relationship between miRNA and mRNA as the final miRNA-mRNA network considering the specific role of miRNA in degrading their target mRNAs (31, 32). Results were visualized using the Cytoscape software (v3.8.0). To investigate a comprehensive set of functional annotation of target mRNAs in networks of obesity, Kyoto Encyclopedia of Genes and Genomes (KEGG) pathway analyses (33) and gene ontology (GO) term enrichment analyses (34) were performed by using the Clusterprofiler package. The KEGG pathway and GO enrichment analyses of genes were based on the threshold of P‐value < 0.05.

### Identifying DE mRNAs of ccRCC From miRNA-mRNA Networks of Obesity

Target mRNAs of miRNA-mRNA networks obtained from the former step in both VAT and SAT were respectively analyzed in the specific samples from obese participants of GSE46699 dataset. By running Bayesian method in limma R package, we identified the DE mRNAs of ccRCC in obesity with P-value < 0.05, and furthermore screened out those with same trends (up- or down-regulated) as shown in networks of obesity. Thereafter, miRNA-mRNA networks of obesity-related ccRCC in both VAT and SAT were constructed, and visualized using Cytoscape software.

### TCGA Data Collection

The RNA sequencing (RNAseq) data from Illumina HiSeq RNA‐Seq platform and corresponding clinical follow‐up information were downloaded from TCGA (https://portal.gdc.cancer.gov/) database. Fragments Per Kilobase Million (FPKM) values of both mRNA and miRNA data were collected. For mRNA data, we transferred ENSAMBL IDs in the matrix file into gene symbols. As a result, 611 and 616 samples were respectively selected for mRNA and miRNA analyses from TCGA database by searching KIRC (kidney renal clear cell carcinoma), while clinical follow-up information were only collected among 537 samples.

### Receiver Operating Characteristic Curve Analysis of RNAs in Networks of Obesity-Related ccRCC

To evaluate the sensitivity and specificity of both mRNAs and miRNAs in networks of obesity-related ccRCC in both VAT and SAT for distinguishing between normal and cancer tissues, ROC curve analyses were respectively conducted for mRNAs and miRNAs, and the area under curve (AUC) was calculated by running the code of “proc logistic” in the SAS software (v9.4). DE RNAs with both statistically significant odds ratios (ORs) and same trends as shown in previous obtained networks were retained to construct the updated networks in VAT and SAT. Sankey diagrams of networks were given using R software. To further verify the relationship of miRNA-mRNA pairs shown in Sankey diagrams, we then performed Spearman correlation analyses focusing on the specific RNAs in networks by selecting samples with both mRNA and miRNA data in TCGA dataset. miRNA-mRNA pairs with inverse correlation coefficients (r < 0 and P-value < 0.05) were considered as final networks.

### Prognostic Signature Analysis of DE RNAs of Obesity-Related ccRCC

To differentiate the ccRCC prognostic signatures of DE RNAs of obesity between VAT and SAT, we performed Cox regression analyses to estimate the association of all DE mRNAs and miRNAs of obesity (which were not limited to those in networks) with the overall survival using the TCGA dataset of ccRCC. 530 and 516 samples with ccRCC were respectively selected for the analyses of mRNAs and miRNAs. Multivariable Cox proportional hazards regression analyses were performed by running SAS code of “proc phreg”, HR and relevant 95% CI were given. Regression models included unadjusted model and multi-adjusted model (adjusted for age, gender, as well as the tumor stage according to the tumor-node-metastasis (TNM) staging system of ccRCC). mRNA and miRNA with both statistical significant HRs (P-value < 0.05) and same trends as shown in previously performed DE analyses were then remained as candidates to calculate a risk score for each patient using regression coefficients of these eligible RNAs derived from Cox regression models. The formula was shown as follows: Risk score= expRNA1 * βRNA1 + expRNA2 * βRNA2 + expRNA3 * βRNA3 +……+ expRNAn * βRNAn, expRNA implies the expression value of RNA, and βRNA implies the regression coefficient of Cox regression model (17). In accordance with the risk scores, patients were divided into high- and low-risk groups, and 1-year ROC as well as Kaplan-Meier survival curves of the two groups were generated by running “timeROC”, “survival”, and “surviminer” R packages. Simultaneously, scatter plots of risk scores and survival time among patients, as well as heatmaps of RNA components used for calculating the risk score were given using R software.

## Results

### Identification of DE mRNAs of Obesity in VAT and SAT

**Figure 2:**
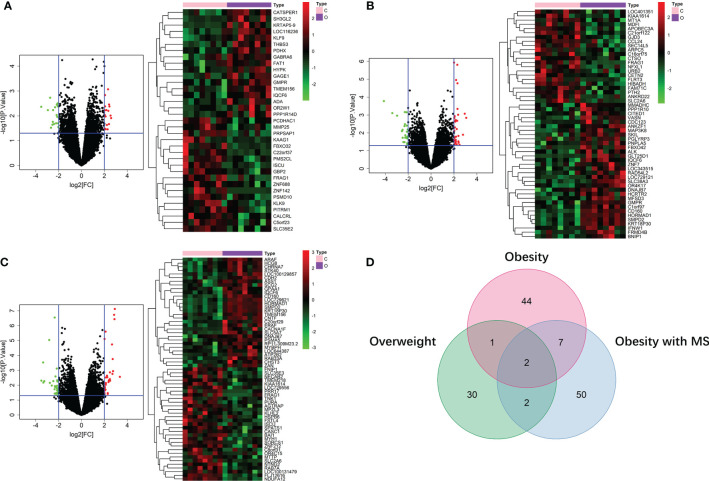
DE mRNAs of overweight **(A)**, obesity **(B)**, and obesity with MS **(C)** in VAT. The red points in the volcano plots represent upregulation and the green plots represent downregulation. The color in heatmaps from green to red shows the progression from low expression to high expression. The amount of DE mRNAs of obesity in VAT was 136 (35 for overweight, 54 for obesity, and 61 for obesity with MS). Venn diagram **(D)** shows that only 2 mRNAs (IQCF6 and FRAG1) were shared by all these 3 obesity components.

**Figure 3:**
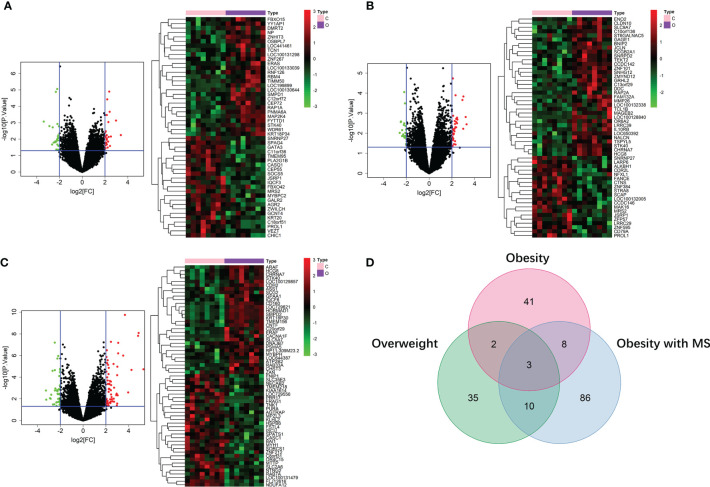
DE mRNAs of overweight **(A)**, obesity **(B)**, and obesity with MS **(C)** in SAT. The red points in the volcano plots represent upregulation and the green plots represent downregulation. The color in heatmaps from green to red shows the progression from low expression to high expression. The amount of DE mRNAs of obesity in VAT was 185 (50 for overweight, 54 for obesity, and 107 for obesity with MS). Venn diagram **(D)** shows that only 3 mRNAs (JSRP1, MRS2 and STK40) were shared by all these 3 obesity components.

By running limma R package among 64 samples in the dataset of GSE24833, we obtained DE mRNAs of overweight, obesity and obesity with MS in both VAT and SAT (**Supplemental Tables 1** and **2**). Volcano plots and heatmaps of DE mRNAs in VAT and SAT were respectively shown in **Figure 2** and **Figure 3**. The amount of DE mRNAs of obesity in VAT was 136 (35 for overweight, 54 for obesity, and 61 for obesity with MS), and only 2 mRNAs (IQCF6 and FRAG1) were shared by all these 3 obesity components as shown in Venn diagram (**Figure 2D**). For SAT, the amount of DE mRNAs of obesity was 185 (50 for overweight, 54 for obesity, and 107 for obesity with MS), and only 3 mRNAs (JSRP1, MRS2 and STK40) were shared by all these 3 obesity components as shown in Venn diagram (**Figure 3D**).

### Construction of miRNA-mRNA Networks of Obesity in VAT and SAT

**Figure 4:**
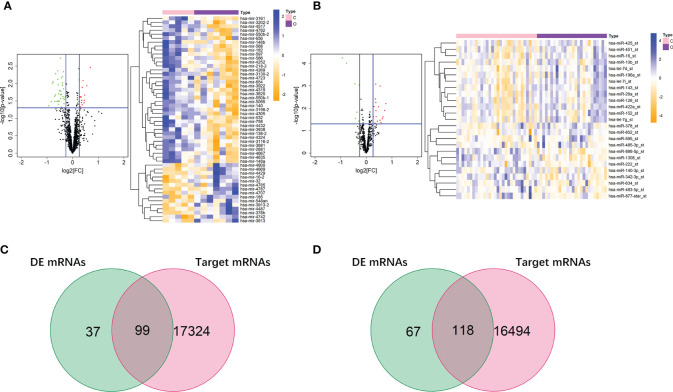
DE miRNAs of obesity in VAT and SAT. 52 DE miRNAs of obesity were screened out in VAT **(A)**, and 25 were screened out in SAT **(B)**. The red points in the volcano plots represent upregulation and the green plots represent downregulation. The color in heatmaps from blue to yellow shows the progression from low expression to high expression. Venn diagrams **(C, D)** shows that the intersections of DE mRNAs of obesity and predicted target mRNAs in miRNA-mRNA pairs were 99 and 118 for VAT **(C)** and SAT **(D)**, respectively.

In the dataset of GSE50574, we screened out 52 DE miRNAs of obesity in VAT by comparing 7 obese to 5 lean samples. On the other hand, 25 miRNAs of obesity in SAT were obtained in the dataset of GSE25402. Values of |log2FC| and P were shown in **Supplemental Table 3**. Volcano plots and heatmaps of DE miRNAs of obesity in VAT and SAT were shown in **Figures 4A, B**. According to the miRNA-mRNA pairs predicted using Targetscan database, 17,423 and 16,612 target mRNAs were respectively obtained for 52 DE miRNA of obesity in VAT and 25 in SAT. By performing VennDiagram R package, the intersections of DE mRNAs of obesity and predicted target mRNAs in miRNA-mRNA pairs were 99 (**Figure 4C**) and 118 (**Figure 4D**) for VAT and SAT, respectively. By further screening out inverse miRNA-mRNA pairs including mRNAs in the intersection, the networks of obesity in VAT and SAT were constructed as shown in **Supplemental Figure 1**. In the network of VAT, 87 mRNAs and 27 miRNAs were included. In the network of SAT, 101 mRNAs and 16 miRNAs were included.

### Functional Annotation of DE mRNAs in Networks of Obesity in VAT and SAT

To gain insights into the different biological features of miRNA-mRNA networks of obesity in VAT and SAT, KEGG and GO analyses were respectively performed for target mRNAs. As shown in **Table 1**, target mRNAs in the miRNA-mRNA network of obesity in VAT were enriched in 6 pathways as following: nicotine addiction (hsa05033; genes: CHRNA7 and GABRA6); cholinergic synapse (hsa04725; genes: CACNA1F, CHRNA7, and SLC5A7); vascular smooth muscle contraction (hsa04270; genes: ARAF, CACNA1F, and CALCRL); mineral absorption (hsa04978; genes: ATP2B2 and MT1A); non-small cell lung cancer (hsa05223; genes: ALK and ARAF); arrhythmogenic right ventricular cardiomyopathy (hsa05412; genes: CACNA1F and CDH2). For the network of SAT, target mRNAs were enriched in 4 pathways as following: pancreatic secretion (hsa04972; genes: ATP1A2, PLA2G1B, and RAP1A); synthesis and degradation of ketone bodies (hsa00072; genes: HMGCS2); PPAR signaling pathway (hsa03320; genes: ACSL4 and HMGCS2); RNA degradation (hsa03018; genes: ENO2 and WDR61). Results of GO enrichment analyses were shown in **Supplemental Figure 2**. In brief, biological process (BP) functional enrichment found that target mRNAs of network of obesity in VAT were enriched in functions like cellular response to glucocorticoid stimulus, cellular response to corticosteroid stimulus, and eosinophil chemotaxis. Target mRNAs in SAT were enriched in functions such as cellular response to drug, DNA methylation or demethylation, and regulation of histone H3-K9 acetylation.

**Table 1:** KEGG enrichments of target mRNAs in miRNA-mRNA networks of obesity in VAT and SAT.

ID	Description	GeneRatio	P-value	geneID	Count
**VAT**					
hsa05033	Nicotine addiction	2/37	0.014244	CHRNA7/GABRA6	2
hsa04725	Cholinergic synapse	3/37	0.014618	CACNA1F/CHRNA7/SLC5A7	3
hsa04270	Vascular smooth muscle contraction	3/37	0.022488	ARAF/CACNA1F/CALCRL	3
hsa04978	Mineral absorption	2/37	0.029596	ATP2B2/MT1A	2
hsa05223	Non-small cell lung cancer	2/37	0.042611	ALK/ARAF	2
hsa05412	Arrhythmogenic right ventricular cardiomyopathy	2/37	0.048094	CACNA1F/CDH2	2
**SAT**					
hsa04972	Pancreatic secretion	3/36	0.010288	ATP1A2/PLA2G1B/RAP1A	3
hsa00072	Synthesis and degradation of ketone bodies	1/36	0.043658	HMGCS2	1
hsa03320	PPAR signaling pathway	2/36	0.044704	ACSL4/HMGCS2	2
hsa03018	RNA degradation	2/36	0.04793	ENO2/WDR61	2

KEGG, Kyoto Encyclopedia of Genes and Genomes; VAT, visceral adipose tissue; SAT, subcutaneous adipose tissue.

### Construction of miRNA-mRNA Networks of Obesity-Related ccRCC in VAT and SAT

**Figure 5:**
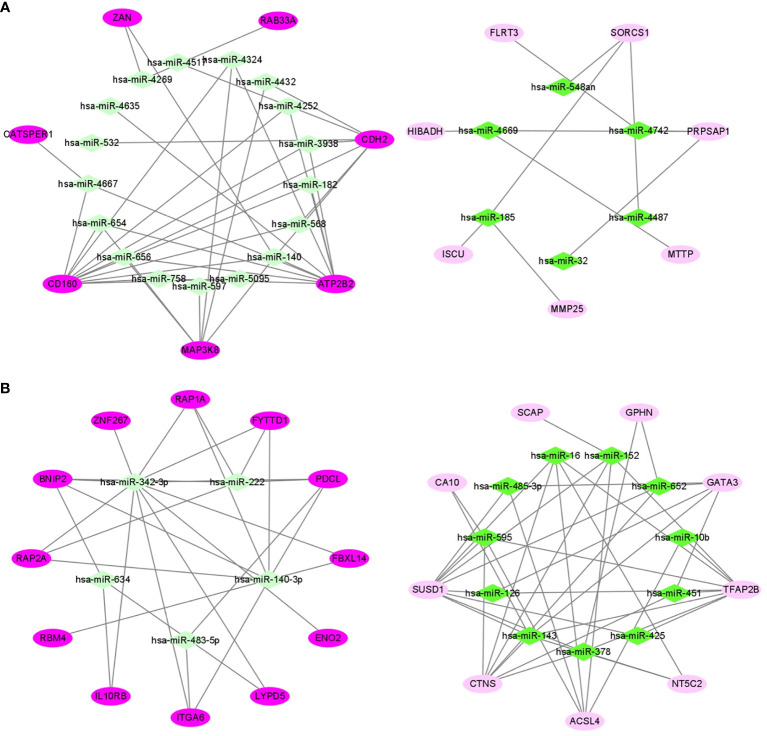
miRNA-mRNA networks of obesity-related ccRCC in VAT **(A)** and SAT **(B)**. In VAT **(A)**, 14 mRNAs and 23 miRNAs were included. In SAT **(B)**, 21 mRNAs and 16 miRNAs were included. Green diamonds represent miRNAs, and pink ellipses represent mRNAs. The color from dark to light respectively represents up- and down-regulated.

In 24 samples (patient-matched tumors and adjacent-normal tissues) from 12 obese participants the dataset of ccRCC (GSE46699), we screened out 28 DE mRNAs of ccRCC (P-value < 0.05) among 87 target mRNAs in the network of obesity in VAT (**Supplemental Table 4**). By further selecting those with same trends (up- or down-regulated) as shown in the network of obesity, 14 mRNAs were retrained. In accordance with these 14 mRNAs, a miRNA-mRNA network of obesity-related ccRCC in VAT was constructed and visualized as **Figure 5A**. In this network, 23 miRNAs were included. Consistent methods were repeated among 101 target mRNAs in the network of obesity in SAT, and 21 mRNAs were finally retrained. By using these 21 mRNAs and 16 paired miRNAs, the miRNA-mRNA network of obesity-related ccRCC in SAT was constructed (**Figure 5B**).

### Verification of miRNA-mRNA Networks of Obesity-Related ccRCC Using TCGA Dataset

**Figure 6:**
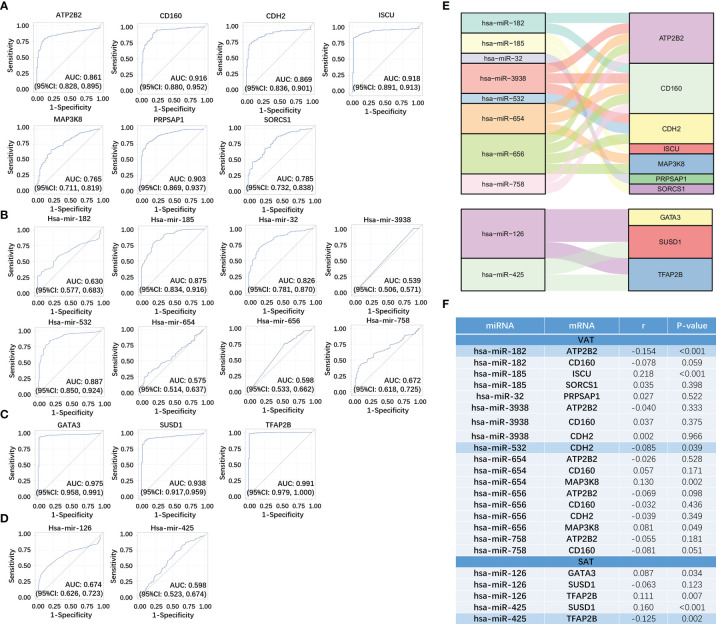
Verification of miRNA-mRNA networks of obesity-related ccRCC using TCGA dataset. For VAT, 7 mRNA **(A)** and 8 paired miRNAs **(B)** with statistically significant ORs were retrained. For SAT, 3 mRNA **(C)** and 2 paired miRNAs **(D)** with statistically significant ORs were retrained. miRNA-mRNA networks of obesity-related ccRCC were visualized using Sankey Diagram **(E)**. Spearman correlation analyses **(F)** found that 3 miRNA-mRNA pairs showed inverse relationship with statistically significant correlation coefficients, including hsa-miR-182&ATP2B2 in VAT, hsa-miR-532&CDH2 in VAT, and hsa-miR-425&TFAP2B in SAT.

We respectively selected 611 samples (including 72 controls) and 616 samples (including 71 controls) in the TCGA dataset of ccRCC to evaluate the sensitivity and specificity of both mRNAs and miRNAs in networks of obesity-related ccRCC. Among 14 target mRNAs and 23 miRNAs in the network of obesity-related ccRCC in VAT, 13 mRNAs and 8 miRNAs with same trends as shown in the network had statistically significant ORs (shown in **Supplemental Table 5**). Seven of 13 mRNAs that could be paired with 8 selected miRNAs were retrained, and ROCs were shown in **Figures 6A, B**. By performing consistent methods, 17 mRNAs and 2 miRNAs were respectively selected from 21 target mRNAs and 16 miRNAs in the network of obesity-related ccRCC in SAT, ORs and 95%CI were shown in **Supplemental Table 5**. Among 17 mRNAs, 3 were found to be paired with 2 miRNAs, and ROCs of them were shown in **Figures 6C, D**. Sankey diagrams of miRNA-mRNA networks were shown in **Figure 6E**, 18 and 5 miRNA-mRNA pairs in both VAT and SAT were included. Furthermore, 588 samples (71 controls) with both mRNA and miRNA data in TCGA dataset were selected to perform Spearman correlation analyses among these RNA pairs. After adjusted for group (tumor or adjacent tissue), 3 pairs showed inverse relationship with statistically significant correlation coefficients, including hsa-miR-182&ATP2B2 (r = -0.154, P-value < 0.001) in VAT, hsa-miR-532&CDH2 (r = -0.085, P-value =0.039) in VAT, and hsa-miR-425&TFAP2B (r = -0.125, P-value =0.002) in SAT (**Figure 6F**). By searching these 3 target mRNAs in GSE24833, all of them were DE mRNAs of obesity with MS.

### Construction of Prognostic Signature Based on DE RNAs of Obesity-Related ccRCC

We firstly screened out the DE RNAs (mRNAs and miRNAs) of ccRCC among DE RNAs of obesity. From 136 DE mRNAs and 52 DE miRNAs of obesity in VAT (shown in **Figures 2** and **3A**), 17 DE mRNAs and 44 DE miRNAs of ccRCC were respectively selected using the TCGA dataset. For DE RNAs of obesity in SAT, 24 mRNAs and 11 miRNAs were finally remained. Multivariable Cox regression analyses were performed among these eligible RNAs, crude and adjusted HRs for each RNA were shown in **Supplemental Table 6**. As results showed, 5 DE mRNAs (SORCS1, FLRT3, HIBADH, CATSPER1, and MAP3K8) and 5 DE miRNAs (hsa-miR-3130-2, hsa-miR-148b, hsa-miR-2681, hsa-miR-4487, and hsa-miR-3613) of obesity-related ccRCC with statistically significant adjusted HRs remained consistent trends (up- and down-regulated) as shown in DE analyses of obesity in VAT. For DE RNAs of obesity-related ccRCC in SAT, 4 mRNAs (ENO2, YY1AP1, FAP, IL10RB) and 1 miRNA (hsa-miR-425) were eligible for the following risk score analyses. By utilizing adjusted regression coefficients of these RNAs, we calculated risk scores of both mRNAs and miRNAs for each patient. Formulas were shown as following:


$$\begin{array}{l} \mathrm{Risk score of mRNAs in VAT}=\text{SORCS}1*(-0.936)+\text{FLRT}3*(-0.055)+\text{ HIBADH}*(0.016)+\text{CATSPER}1*(0.832)+\text{MAP}3\text{K}8*(0.133); \end{array}$$

Risk score of miRNAs in VAT=hsa−miR−3130−2∗(−0.200)+hsa−miR−148b∗(0.335)+ hsa−miR−2681∗(−0.519)+hsa−miR−4487∗(0.628)+hsa−miR−3613∗(0.268);

Risk score of mRNAs in SAT=ENO2∗(0.006)+YY1AP1∗(0.090)+ FAP+(0.113)+IL10RB∗(0.036)

**Figure 7:**
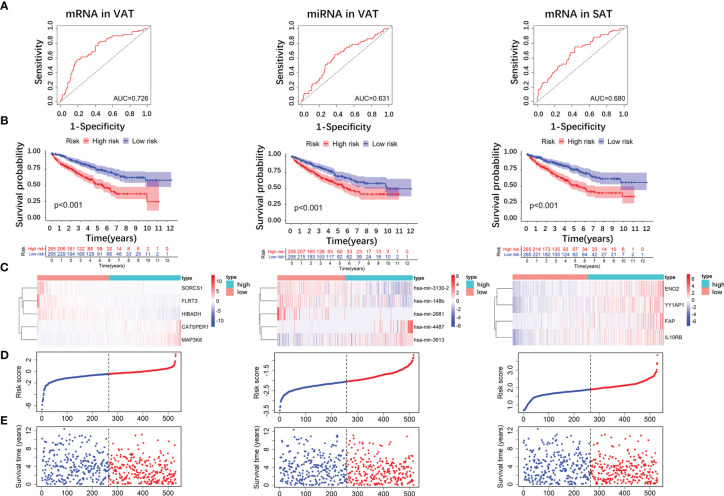
Risk score analyses of DE RNAs of obesity-related ccRCC in the prognosis of ccRCC. 5 DE mRNAs (SORCS1, FLRT3, HIBADH, CATSPER1, and MAP3K8), 5 DE miRNAs (hsa-miR-3130-2, hsa-miR-148b, hsa-miR-2681, hsa-miR-4487, and hsa-miR-3613) of obesity-related ccRCC in VAT, and 4 mRNAs (ENO2, YY1AP1, FAP, IL10RB) in SAT were used to calculate risk scores. AUC of risk score of mRNAs in VAT was larger than those of miRNAs in VAT, and mRNAs in SAT **(A)**. Overall survival time of patients in the low‐risk group was significantly longer than in the high‐risk group regardless of risk score models **(B)**. Distributions of RNA expressions, risk scores, and survival statuses for each patient were also shown **(C–E)**.

We divided patients into high- and low-risk groups according to the medians of risk scores. As shown in **Figure 7A**, AUCs of risk score models were 0.726 (mRNAs in VAT), 0.631 (miRNAs in VAT), and 0.680 (mRNAs in SAT). In addition, overall survival time of patients in the low‐risk group was significantly long than that in the high‐risk group regardless of risk score models (P-values < 0.001, **Figure 7B**). Distributions of RNA expressions, risk scores, and survival statuses for each patient were shown in **Figures 7C–E**.

## Discussion

In recent years, obesity has become a worldwide health problem. Emerging evidence shows that obesity is a risk factor of various non-communicable diseases, including cancers (35), and main mechanisms could be partially attributed to the inflammation triggered by excess adipose tissue. Abdominal adipose tissue is a major storage of body fat, consisting of VAT and SAT. Differences in abdominal locations, structural compositions, metabolic activities, and functional significances between VAT and SAT implied that their pathophysiological mechanisms might be different (36), while no study has disclosed such heterogeneity. In our study, 136 and 185 DE mRNAs of obesity were respectively found in VAT and SAT using GEO datasets, while no RNA was overlapped between these two adipose tissues. DE genes between VAT and SAT have been reported in several published studies (37–39), our result was in line with previous findings, and implied that VAT and SAT perhaps differed in pathophysiological functions. Simultaneously, in accordance with miRNA datasets of GEO, different miRNA-mRNA regulatory networks of obesity in VAT and SAT were obtained. To further explore the heterogeneity in biological functions between VAT and SAT, KEGG and GO analyses were performed among mRNAs in obtained miRNA-mRNA networks of obesity. As we found, 6 and 4 non-overlapped biological pathways were respectively enriched for mRNAs in networks of both VAT and SAT. Results from enrichment analyses indicated that miRNA-mRNA regulatory networks we screened out were biologically functional, and also disclosed different pathophysiological mechanisms of obesity-related diseases potentially existing in VAT and SAT.

The association of ccRCC with excessively accumulated adipose tissue has been revealed in several studies. In a newly published study, both VAT and SAT areas measured using CT were found to be positively associated with ccRCC among Caucasian patients with different genetic background (40). There is a multi-center study from Chinese population showing that the mean VAT area measured by CT in ccRCC group was larger than that in non-ccRCC group by 25 cm^2^, and such difference was statistically significant (41). In another study, statistical differences of both VAT and SAT were found between ccRCC cases and controls, and smaller P-value (<0.001) for VAT was shown (13). Additionally, it has been reported that individuals with high levels of C-peptide, interleukin-6 (IL-6), tumor necrosis factor-α (TNF-α), as well as low level of adiponectin had significantly higher risk of ccRCC, all these biomarkers are obesity-related (8, 42). Even though associations of ccRCC with both biochemical and image-based indices of obesity have been estimated, relevant bioinformatic studies still remained limited. Zhou, et al. performed a data-mining study by combining GEO with TCGA datasets, and 5 genes correlated with both ccRCC and obesity were found (IGHA1 and IGKC were oncogenes, and MAOA, MUC20 and TRPM3 were tumor suppressor genes) (19). However, if RNAs regarding to obesity-related ccRCC were different between VAT and SAT still remained underdetermined. On the other hand, miRNAs from both plasma and tissue samples have been reported to be associated with ccRCC (22, 43), while miRNA-mRNA regulatory networks of obesity-related ccRCC were not given in the above mentioned bioinformatic study.

In our study, based on the obtained miRNA-mRNA networks of obesity in VAT and SAT, we only included samples of obese participants from the GEO dataset of ccRCC, and screened out specific networks of obesity-related ccRCC (14 mRNAs and 23 miRNAs were included in the network of VAT; 21 mRNAs and 16 miRNAs were included in the network of SAT). After further verified using TCGA dataset, 18 and 5 miRNA-mRNA pairs were respectively screened out for VAT and SAT, and only 3 pairs remained inverse relationship in Spearman correlation analyses (hsa-miR-182&ATP2B2, hsa-miR-532&CDH2 in VAT; and hsa-miR-425&TFAP2B in SAT). All these 3 miRNAs were shown to be associated with ccRCC, despite that reported target genes distinguished from those we found. For example, hsa-miR-182, and hsa-miR-532 could suppresses RCC migration and proliferation respectively *via* targeting insulin-like growth factor 1 receptor (IGF1R), or nucleosome assembly protein 1-like 1 (NAP1L1) (44, 45). In contrast, hsa-miR-425 could perform an oncogenic effect by promoting invasion and migration of RCC cell lines (46). Although all 3 genes of ATP2B2, CDH2 and TFAP2B have been reported to play a role in pathophysiology of various cancers (47–49), reports referring to the association with RCC remained limited. Zhang, et al. found that the elevation of CDH2 could promote growth, migration, and invasion abilities of RCC cells (49). According to the published evidence as above mentioned, all these 3 miRNA-mRNA pairs we screened out in VAT and SAT perhaps could play some potential roles in the pathogenesis of obesity-related ccRCC, while the following experimental studies verifying their regulatory relationships are still necessary.

Even though obesity have been generally identified as a risk factor of ccRCC, the paradox between obesity and ccRCC prognosis was still frequently disclosed (22, 50, 51). Sanchez, et al. combined 3 cohort studies (COMPARZ trial, TCGA cohort, and MSK immunotherapy study) and obtained the consistent results of survival advantage in obese patients with ccRCC (51). Findings from this study implied that the peritumoral adipose tissue might contribute to the better prognosis of ccRCC by acting as an immune reservoir. However, the potential roles of abdominal adipose tissue, as the main storage of fat, in the prognosis of ccRCC still remained unknown. Based on previously published findings showing the excess accumulated VAT and SAT were positively associated with the prevalence of ccRCC (13, 41), as well as what we found in this current study, we hypothesized that there probably could exist some DE RNAs of obesity-related ccRCC in VAT and SAT remaining consistent performance in terms of ccRCC prognosis, i.e. up-regulated DE RNAs of obesity-related ccRCC could be correlated with poor prognosis, and down-regulated ones could be correlated with better prognosis. As we found in the survival analyses, 5 DE mRNAs (SORCS1, FLRT3, HIBADH, CATSPER1, and MAP3K8) and 5 DE miRNAs (hsa-miR-3130-2, hsa-miR-148b, hsa-miR-2681, hsa-miR-4487, and hsa-miR-3613) of obesity-related ccRCC were found in VAT. For DE RNAs of obesity in SAT, 4 mRNAs (ENO2, YY1AP1, FAP, IL10RB) and 1 miRNA (hsa-miR-425) were found. Most of selected RNAs were reported to be relevant to prognosis of various cancers, while studies referring to their associations with RCC prognosis are still limited. Sun, et al. found that ENO2, i.e. enolase 2, was associated with worsened prognosis in papillary RCC and was related to glycolysis (52). hsa-miR-3613 and hsa-miR-425 were also shown to be associated with poor prognosis of ccRCC and chromophobe RCC, respectively (53, 54). Our subsequent risk score analyses by utilizing above selected RNAs also found that patients in the low‐risk group survived significantly longer than those in the high‐risk group regardless of risk score models. Findings in our survival analyses implied that there probably existed some potential mechanisms underlying the association between obesity and poor prognosis of ccRCC. However, factors correlated with ccRCC prognosis were complicated, and biological functions of relevant RNAs found in this study should be further verified.

Cautions should be paid when interpreting our findings. Firstly, although DE RNAs of obesity were estimated based on the adipose tissue samples (VAT or SAT), both GEO and TCGA datasets of ccRCC consist of samples only from tumor or adjacent tissues. Therefore, some RNAs only highly expressed in adipose tissue but not in tumor tissues perhaps could be eliminated. However, it was still reasonable to consider that the eventually selected DE RNAs of obesity-related ccRCC might have some potential pathophysiological effects underlying the association between obesity and ccRCC. Further studies directly focusing on the selected RNAs from adipose tissues of both ccRCC cases and controls would be necessary. Secondly, there are several noncoding RNAs such as circular RNA (circRNA) and long non-coding RNA (lncRNA), which could compete for miRNA binding, and thereafter regulate the expression of mRNA. However, we only constructed the miRNA-mRNA networks in this study since that datasets of circRNA or lncRNA cannot be simultaneously found for both VAT and SAT. Thirdly, only ccRCC was considered in our study, while the biological functions of VAT and SAT might be different in terms of other subtypes of renal cancer. The association of DE RNAs and miRNA-mRNA networks selected in our study with other renal cancers should be further estimated.

## Conclusions

In this study, we respectively constructed specific miRNA-mRNA regulatory networks of obesity-related ccRCC for both VAT and SAT, and 3 RNA pairs (i.e. hsa-miR-182&ATP2B2, hsa-miR-532&CDH2 in VAT; and hsa-miR-425&TFAP2B in SAT) were finally screened out. This finding indicated that VAT and SAT might perform distinct functions in the pathogenesis of obesity-related ccRCC. On the other hand, several DE RNAs of obesity-related ccRCC were found to remain consistent performance in terms of ccRCC prognosis, implying that there probably existed some potential mechanisms underlying the association between obesity and poor prognosis of ccRCC. What we found disclosed heterogeneous molecular mechanisms between VAT and SAT in relation to the prevalence and prognosis of ccRCC, and could provide valuable evidence on the targeted therapy of obesity-related ccRCC.

## Data Availability Statement

The datasets presented in this study can be found in online repositories. The names of the repository/repositories and accession number(s) can be found in the article/**Supplementary Material**.

## Author Contributions

YuL and YM: conception and design. YuL: development of methodology. YuL and YM: analysis and interpretation of data. YuL: writing of the manuscript. YaL, JH, and LL: review of the manuscript. DW: study supervision. All authors contributed to the article and approved the submitted version.

## Funding

This research was supported by the National Key R&D Program of China [Grant#2018YFC1311600].

## Conflict of Interest

The authors declare that the research was conducted in the absence of any commercial or financial relationships that could be construed as a potential conflict of interest.

## Publisher’s Note

All claims expressed in this article are solely those of the authors and do not necessarily represent those of their affiliated organizations, or those of the publisher, the editors and the reviewers. Any product that may be evaluated in this article, or claim that may be made by its manufacturer, is not guaranteed or endorsed by the publisher.

## Supplementary Material

Supplementary Figure 1 miRNA-mRNA networks of obesity in VAT **(A)** and SAT **(B)**. In the network of VAT **(A)**, 87 mRNAs and 27 miRNAs were included. In the network of SAT **(B)**, 101 mRNAs and 16 miRNAs were included. Blue and green ellipses represent mRNAs, and pink diamonds represent miRNAs. The color from dark to light respectively represents up- and down-regulated.

Supplementary Figure 2 Results of GO enrichment analyses in VAT **(A–C)** and SAT **(D–F)**. **(A, D)**: results of biological process (BP) functional enrichment in mRNAs of VAT **(A)** and SAT **(D)**; **(B, E)**: results of molecular function (MF) functional enrichment in mRNAs of VAT **(B)** and SAT **(E)**; **(C, F)**: results of cellular component (CC) functional enrichment in mRNAs of VAT **(C)** and SAT **(F)**. The color from red to blue represents p-values.
