# Supplementary figures and images for: Heterogeneous miRNA-mRNA Regulatory Networks of Visceral and Subcutaneous Adipose Tissue in the Relationship Between Obesity and Renal Clear Cell Carcinoma
Source: Front Endocrinol (Lausanne). 2021 Sep 21;12:713357. doi: 10.3389/fendo.2021.713357 (PMC8490801; doi:10.3389/fendo.2021.713357)

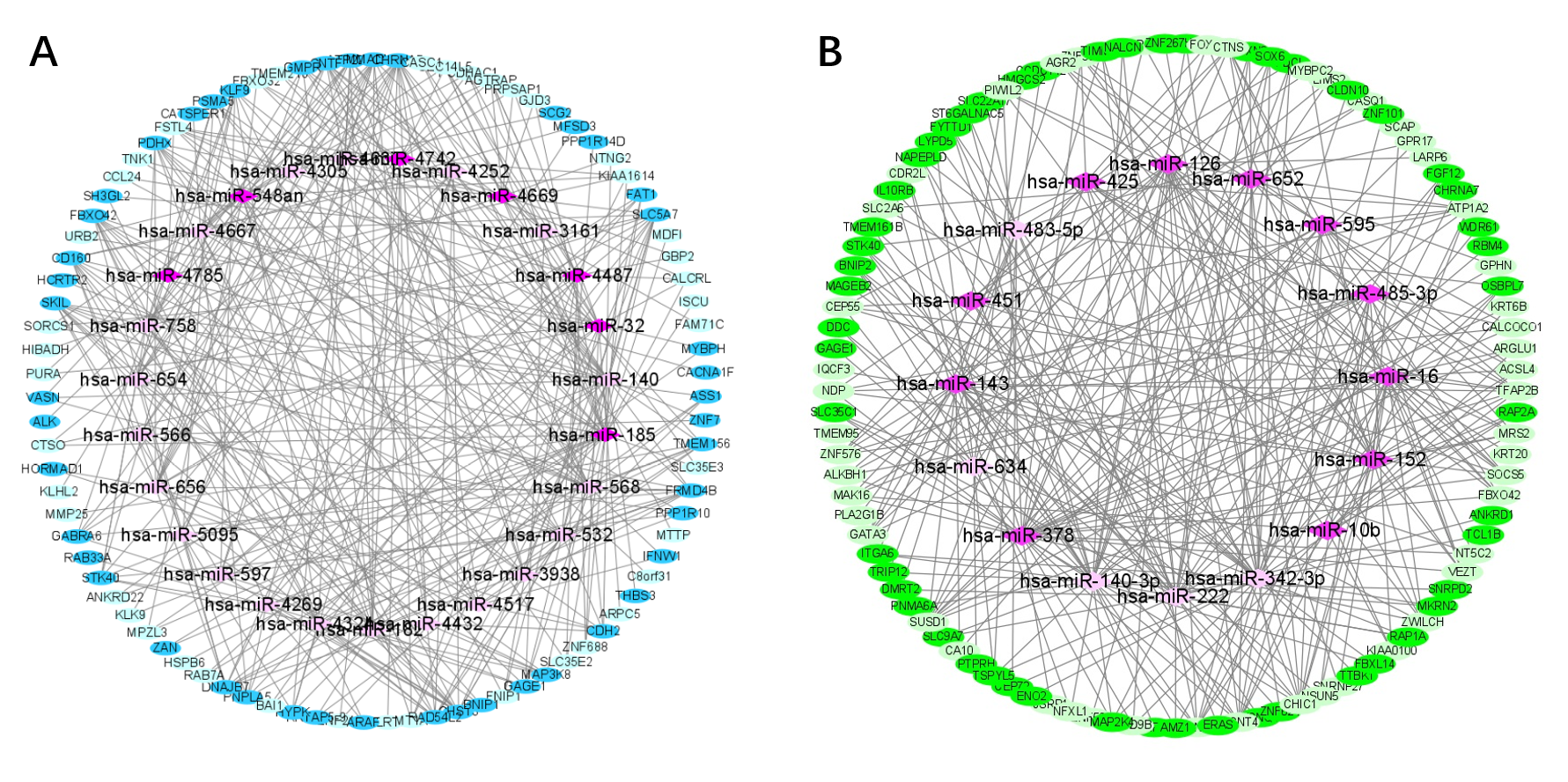

Supplement: Supplementary Figure 1 — miRNA-mRNA networks of obesity in VAT (A) and SAT (B). In the network of VAT (A), 87 mRNAs and 27 miRNAs were included. In the network of SAT (B), 101 mRNAs and 16 miRNAs were included. Blue and green ellipses represent mRNAs, and pink diamonds represent miRNAs. The color from dark to light respectively represents up- and down-regulated. [file Image_1.tif]

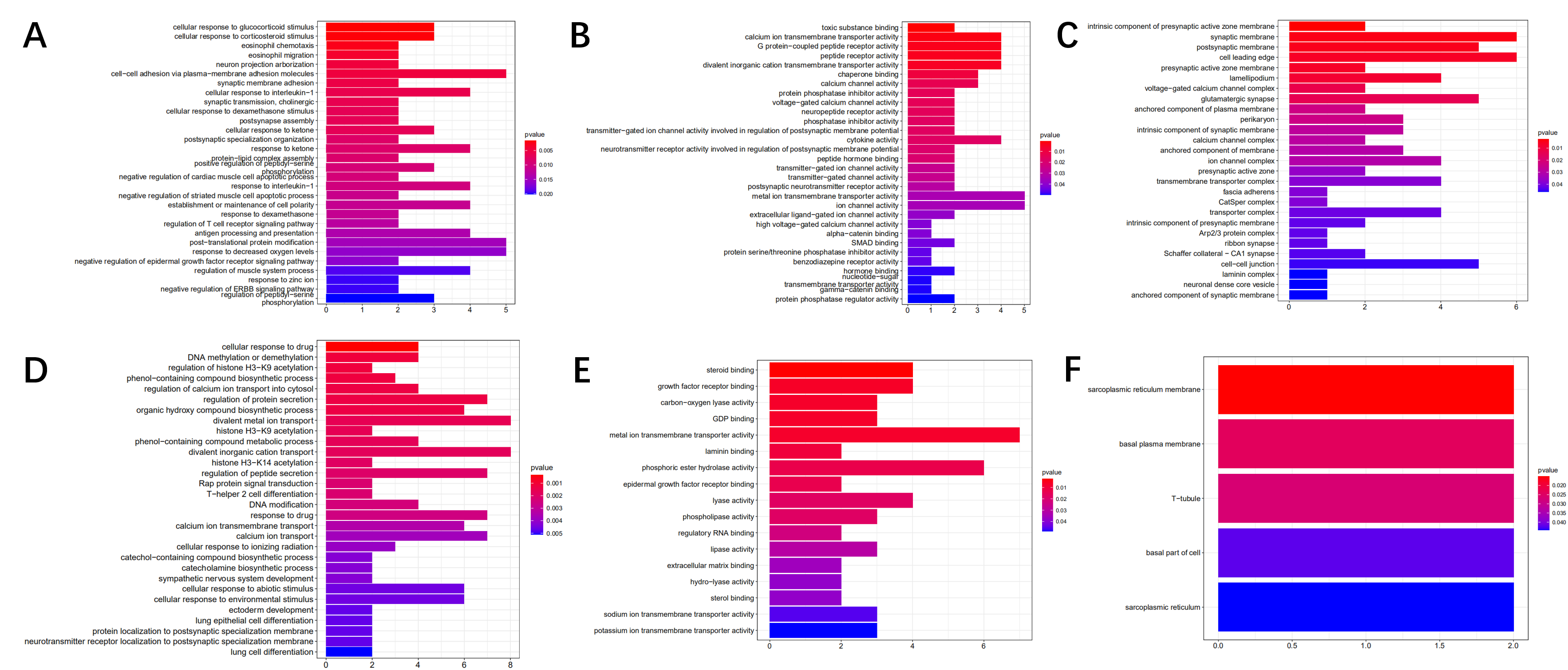

Supplement: Supplementary Figure 2 — Results of GO enrichment analyses in VAT (A–C) and SAT (D–F). (A, D): results of biological process (BP) functional enrichment in mRNAs of VAT (A) and SAT (D); (B, E): results of molecular function (MF) functional enrichment in mRNAs of VAT (B) and SAT (E); (C, F): results of cellular component (CC) functional enrichment in mRNAs of VAT (C) and SAT (F). The color from red to blue represents p-values. [file Image_2.tif]
