# Supplementary material for: Heterogeneous miRNA-mRNA Regulatory Networks of Visceral and Subcutaneous Adipose Tissue in the Relationship Between Obesity and Renal Clear Cell Carcinoma
Source: Front Endocrinol (Lausanne). 2021 Sep 21;12:713357. doi: 10.3389/fendo.2021.713357 (PMC8490801; doi:10.3389/fendo.2021.713357)
Supplement: Supplementary file 3 [file DataSheet_1.docx]

**No. of supplemental tables and figures:** 6 tables and 2 figures

| **Supplemental table1** Differentially expressed mRNA of overweight, obesity, and obesity with metabolic syndrome in VAT | | | | | |
| --- | --- | --- | --- | --- | --- |
| **mRNA** | **logFC** | **AveExpr** | **T-value** | **P-value** | **adj.P.Val** |
| **Overweight** |  |  |  |  |  |
| CATSPER1 | 2.318 | 2.900 | 4.048 | 0.001 | 0.644 |
| LOC116236 | 2.053 | 1.501 | 3.799 | 0.001 | 0.644 |
| ISCU | -2.738 | 1.339 | -3.686 | 0.002 | 0.644 |
| PRPSAP1 | -2.067 | 2.019 | -3.445 | 0.003 | 0.644 |
| PSMD10 | -2.104 | 0.090 | -3.434 | 0.003 | 0.644 |
| KLF9 | 2.407 | 0.874 | 3.396 | 0.003 | 0.644 |
| ZNF142 | -2.157 | 1.013 | -3.385 | 0.004 | 0.644 |
| SLC35E2 | -2.068 | -0.178 | -3.314 | 0.004 | 0.644 |
| PDHX | 2.056 | 0.397 | 3.314 | 0.004 | 0.644 |
| IQCF6 | 2.440 | -0.069 | 3.311 | 0.004 | 0.644 |
| FRAG1 | -3.518 | 2.803 | -3.296 | 0.004 | 0.644 |
| C22orf37 | -2.311 | 1.429 | -3.277 | 0.004 | 0.649 |
| KRTAP5-9 | 2.168 | 1.284 | 3.154 | 0.006 | 0.655 |
| FBXO32 | -2.934 | 1.116 | -3.153 | 0.006 | 0.655 |
| PMS2CL | -2.479 | -0.478 | -3.152 | 0.006 | 0.655 |
| THBS3 | 2.020 | 1.573 | 3.142 | 0.006 | 0.655 |
| GMPR | 2.342 | -0.443 | 3.122 | 0.006 | 0.655 |
| TMEM156 | 2.018 | 0.559 | 2.949 | 0.009 | 0.657 |
| GBP2 | -2.177 | -0.217 | -2.939 | 0.009 | 0.657 |
| SH3GL2 | 2.536 | 1.189 | 2.913 | 0.010 | 0.657 |
| ADA | 2.102 | 0.732 | 2.874 | 0.011 | 0.657 |
| PITRM1 | -2.120 | -0.266 | -2.855 | 0.011 | 0.657 |
| HYPK | 2.584 | 1.111 | 2.802 | 0.012 | 0.657 |
| MMP25 | -2.503 | -0.108 | -2.635 | 0.017 | 0.657 |
| KAAG1 | -2.215 | 0.703 | -2.573 | 0.020 | 0.657 |
| PCDHAC1 | -2.042 | 0.010 | -2.569 | 0.020 | 0.657 |
| C5orf23 | -2.401 | -0.735 | -2.539 | 0.021 | 0.657 |
| GABRA6 | 2.398 | 2.013 | 2.535 | 0.021 | 0.657 |
| KLK9 | -2.539 | 0.767 | -2.507 | 0.023 | 0.657 |
| GAGE1 | 2.219 | 2.352 | 2.496 | 0.023 | 0.657 |
| ZNF688 | -2.027 | -0.124 | -2.399 | 0.028 | 0.657 |
| CALCRL | -2.663 | 0.041 | -2.369 | 0.030 | 0.657 |
| FAT1 | 2.013 | 2.577 | 2.334 | 0.032 | 0.660 |
| OR2W1 | 2.109 | 1.870 | 2.299 | 0.035 | 0.666 |
| PPP1R14D | 2.284 | 1.892 | 2.295 | 0.035 | 0.666 |
| **Obesity** |  |  |  |  |  |
| CD160 | 2.010 | 2.671 | 7.330 | 0.000 | 0.014 |
| HORMAD1 | 2.294 | 1.810 | 7.170 | 0.000 | 0.014 |
| SMPD2 | 2.191 | 1.998 | 6.132 | 0.000 | 0.065 |
| KRT18P30 | 2.306 | 2.056 | 5.915 | 0.000 | 0.074 |
| FRAG1 | -4.117 | 2.503 | -4.803 | 0.000 | 0.290 |
| LOC729121 | 2.309 | 2.969 | 4.330 | 0.000 | 0.454 |
| C21orf122 | -2.289 | 0.520 | -4.143 | 0.001 | 0.469 |
| HIBADH | -2.132 | 1.168 | -4.128 | 0.001 | 0.469 |
| FLRT3 | -3.378 | 1.178 | -4.083 | 0.001 | 0.469 |
| OR4K17 | 2.940 | 0.371 | 4.043 | 0.001 | 0.469 |
| ANKZF1 | 2.365 | 0.443 | 3.983 | 0.001 | 0.473 |
| CCL24 | -2.965 | 1.424 | -3.928 | 0.001 | 0.477 |
| SLC38A3 | 2.066 | 0.919 | 3.896 | 0.001 | 0.477 |
| IQCF6 | 3.072 | 0.246 | 3.800 | 0.001 | 0.516 |
| HCRTR2 | 2.034 | 1.775 | 3.791 | 0.001 | 0.516 |
| C1orf97 | 2.159 | 2.915 | 3.671 | 0.002 | 0.554 |
| VASN | 2.129 | 1.346 | 3.648 | 0.002 | 0.566 |
| GLT25D1 | 2.284 | 1.137 | 3.623 | 0.002 | 0.566 |
| SKIL | 2.120 | 1.127 | 3.575 | 0.002 | 0.569 |
| SEC14L5 | -2.274 | 2.189 | -3.423 | 0.003 | 0.572 |
| NFXL1 | -2.117 | 1.758 | -3.337 | 0.004 | 0.604 |
| APOBEC3A | -2.315 | -0.264 | -3.309 | 0.004 | 0.609 |
| GMPR | 2.081 | -0.574 | 3.232 | 0.005 | 0.632 |
| PNPLA5 | 2.245 | 0.984 | 3.133 | 0.006 | 0.632 |
| MDFI | -2.036 | 0.849 | -3.104 | 0.006 | 0.632 |
| PTH2 | -2.486 | 4.685 | -3.089 | 0.007 | 0.632 |
| MAP3K8 | 2.037 | 1.741 | 3.030 | 0.008 | 0.632 |
| GJD3 | -2.540 | 0.387 | -3.006 | 0.008 | 0.632 |
| CETN2 | -2.107 | 0.402 | -2.970 | 0.009 | 0.632 |
| FBXO42 | 2.791 | 1.636 | 2.788 | 0.013 | 0.632 |
| URB2 | -2.246 | 0.711 | -2.755 | 0.014 | 0.632 |
| LOC343515 | 2.390 | 0.300 | 2.717 | 0.015 | 0.632 |
| KIAA1614 | -2.025 | 0.702 | -2.684 | 0.016 | 0.632 |
| MFSD3 | 2.183 | 1.705 | 2.669 | 0.016 | 0.632 |
| SLC2A6 | -2.161 | 1.353 | -2.639 | 0.017 | 0.632 |
| BNIP1 | 2.069 | 2.426 | 2.637 | 0.017 | 0.632 |
| ANKRD22 | -2.258 | 1.115 | -2.605 | 0.018 | 0.632 |
| ARPC5 | -2.477 | 1.099 | -2.604 | 0.019 | 0.632 |
| MMADHC | 2.041 | 0.012 | 2.588 | 0.019 | 0.632 |
| MT1A | -2.097 | -0.202 | -2.564 | 0.020 | 0.632 |
| PPP1R10 | 2.158 | 0.312 | 2.469 | 0.024 | 0.638 |
| IFNW1 | 2.011 | 0.786 | 2.448 | 0.026 | 0.649 |
| FRMD4B | 2.052 | 1.067 | 2.430 | 0.026 | 0.656 |
| CDC123 | 2.391 | 0.776 | 2.376 | 0.030 | 0.674 |
| ALK | 2.174 | 0.200 | 2.349 | 0.031 | 0.674 |
| CTSO | -2.418 | 0.413 | -2.329 | 0.032 | 0.675 |
| LOC401351 | -2.315 | 0.532 | -2.322 | 0.033 | 0.675 |
| CITED1 | 2.130 | 0.381 | 2.305 | 0.034 | 0.675 |
| DNAJB7 | 2.405 | 1.026 | 2.288 | 0.035 | 0.675 |
| FAM71C | -2.084 | 0.382 | -2.252 | 0.038 | 0.683 |
| ZNF7 | 2.103 | 0.574 | 2.217 | 0.041 | 0.687 |
| RAD54L2 | 2.243 | 1.103 | 2.187 | 0.043 | 0.693 |
| C16orf75 | -2.083 | 1.455 | -2.175 | 0.044 | 0.696 |
| PGLYRP3 | 2.485 | 1.619 | 2.153 | 0.046 | 0.696 |
| **Obesity with MS** |  |  |  |  |  |
| HORMAD1 | 2.905 | 2.116 | 9.013 | 0.000 | 0.001 |
| SMPD2 | 2.846 | 2.325 | 8.445 | 0.000 | 0.002 |
| NDUFA12 | -2.345 | 1.968 | -8.189 | 0.000 | 0.002 |
| KRT18P30 | 2.878 | 2.342 | 8.041 | 0.000 | 0.002 |
| CD160 | 2.103 | 2.717 | 6.943 | 0.000 | 0.005 |
| LOC729621 | 2.009 | -0.919 | 6.334 | 0.000 | 0.012 |
| FLJ12616 | -2.820 | 0.370 | -6.252 | 0.000 | 0.013 |
| ARAF | 2.660 | 1.349 | 5.898 | 0.000 | 0.019 |
| CNTF | 2.721 | 1.297 | 4.791 | 0.000 | 0.087 |
| SORCS1 | -3.346 | 1.450 | -4.539 | 0.000 | 0.106 |
| SLC35E3 | -2.160 | 0.527 | -4.099 | 0.001 | 0.151 |
| HCG8 | 2.755 | 1.119 | 3.913 | 0.001 | 0.179 |
| CDH2 | 2.483 | 2.215 | 3.828 | 0.001 | 0.193 |
| STK40 | 2.702 | 1.339 | 3.715 | 0.002 | 0.207 |
| ERAF | 2.221 | 0.646 | 3.593 | 0.002 | 0.227 |
| MYH1 | -2.272 | 1.285 | -3.537 | 0.003 | 0.239 |
| IQCF6 | 3.348 | 0.384 | 3.508 | 0.003 | 0.242 |
| SLC5A7 | 2.249 | 1.722 | 3.478 | 0.003 | 0.244 |
| TMEM156 | 2.127 | 0.613 | 3.458 | 0.003 | 0.244 |
| CHRNA7 | 2.475 | 1.055 | 3.343 | 0.004 | 0.256 |
| AGTRAP | -2.128 | 0.603 | -3.259 | 0.005 | 0.263 |
| GPAA1 | 2.381 | -0.001 | 3.256 | 0.005 | 0.263 |
| PRR17 | -3.505 | 2.441 | -3.234 | 0.005 | 0.263 |
| ZNF212 | -2.068 | 0.641 | -3.230 | 0.005 | 0.263 |
| ATP2B2 | 2.495 | 1.133 | 3.229 | 0.005 | 0.263 |
| FSTL4 | -2.463 | 2.270 | -3.197 | 0.005 | 0.271 |
| NTNG2 | -3.247 | 0.465 | -3.194 | 0.005 | 0.271 |
| NECAB2 | -2.105 | 1.874 | -3.169 | 0.006 | 0.273 |
| PSMA5 | 2.357 | 0.793 | 3.133 | 0.006 | 0.274 |
| FRAG1 | -3.330 | 2.897 | -3.095 | 0.007 | 0.279 |
| FNIP1 | -2.764 | 0.385 | -3.077 | 0.007 | 0.282 |
| LOC100129857 | 2.117 | 0.748 | 3.062 | 0.007 | 0.284 |
| RP11-309M23.2 | 2.134 | 0.156 | 3.058 | 0.007 | 0.285 |
| RAB33A | 2.118 | 0.751 | 2.933 | 0.009 | 0.310 |
| SPATS1 | -2.596 | -0.191 | -2.877 | 0.011 | 0.324 |
| BAI1 | -2.131 | 1.094 | -2.862 | 0.011 | 0.327 |
| RAB7A | -2.316 | 0.590 | -2.836 | 0.011 | 0.333 |
| MPZL3 | -2.310 | 2.025 | -2.781 | 0.013 | 0.347 |
| C20orf29 | 2.046 | 0.733 | 2.759 | 0.014 | 0.352 |
| MTTP | -2.233 | 0.597 | -2.758 | 0.014 | 0.352 |
| CHST3 | 2.001 | 0.983 | 2.717 | 0.015 | 0.361 |
| LOC729556 | -2.104 | 4.169 | -2.680 | 0.016 | 0.373 |
| PURA | -2.145 | 1.699 | -2.675 | 0.016 | 0.373 |
| ASS1 | 2.008 | 0.381 | 2.664 | 0.016 | 0.375 |
| KLHL2 | -2.089 | 1.890 | -2.643 | 0.017 | 0.385 |
| SLC2A6 | -2.044 | 1.411 | -2.629 | 0.018 | 0.386 |
| TMEM218 | -2.352 | 1.628 | -2.603 | 0.019 | 0.393 |
| ZAN | 2.037 | 0.780 | 2.584 | 0.019 | 0.398 |
| MYBPH | 2.094 | 1.366 | 2.576 | 0.020 | 0.400 |
| C8orf31 | -2.138 | 1.511 | -2.529 | 0.022 | 0.406 |
| CACNA1F | 2.052 | 1.090 | 2.521 | 0.022 | 0.406 |
| KIAA1614 | -2.084 | 0.673 | -2.477 | 0.024 | 0.415 |
| ISCU | -2.011 | 1.703 | -2.406 | 0.028 | 0.442 |
| CASC1 | -2.067 | 0.740 | -2.328 | 0.033 | 0.464 |
| LOC100131479 | -2.201 | 1.249 | -2.317 | 0.033 | 0.466 |
| DNAJB7 | 2.290 | 0.968 | 2.307 | 0.034 | 0.467 |
| SCG2 | 2.009 | 3.261 | 2.303 | 0.034 | 0.468 |
| HSPB6 | -2.340 | 1.005 | -2.271 | 0.037 | 0.479 |
| LOC644387 | 2.289 | 0.519 | 2.241 | 0.039 | 0.485 |
| TNK1 | -2.218 | 1.327 | -2.236 | 0.039 | 0.485 |
| OR4C15 | -2.038 | 1.419 | -2.164 | 0.045 | 0.498 |
| **Abbreviation:** VAT: visceral adipose tissue; FC: fold change; AveExpr: average of express level; adj.P.Val: adjusted P-value; MS: metabolic syndrome | | | | | |

| **Supplemental table2** Differentially expressed mRNA of overweight, obesity, and obesity with metabolic syndrome in SAT | | | | | |
| --- | --- | --- | --- | --- | --- |
| **mRNA** | **logFC** | **AveExpr** | **T-value** | **P-value** | **adj.P.Val** |
| **Overweight** |  |  |  |  |  |
| C11orf36 | -2.194 | 1.024 | -6.259 | 0.000 | 0.058 |
| MAP2K4 | 2.376 | 0.877 | 6.070 | 0.000 | 0.058 |
| GATA3 | -2.316 | 3.619 | -6.053 | 0.000 | 0.058 |
| PNMA6A | 2.198 | -0.004 | 5.522 | 0.000 | 0.095 |
| OSBPL7 | 2.411 | 0.162 | 4.683 | 0.000 | 0.357 |
| LOC199899 | 2.160 | 1.006 | 4.468 | 0.000 | 0.398 |
| DMRT2 | 2.971 | 1.227 | 4.110 | 0.001 | 0.503 |
| C18orf51 | -3.379 | 2.970 | -4.042 | 0.001 | 0.519 |
| ZNHIT3 | 2.298 | 2.946 | 4.010 | 0.001 | 0.519 |
| IQCF3 | -2.179 | 0.621 | -3.884 | 0.001 | 0.519 |
| PROL1 | -2.884 | 2.564 | -3.749 | 0.002 | 0.519 |
| SOCS5 | -2.044 | 1.859 | -3.738 | 0.002 | 0.519 |
| TMEM95 | -2.595 | 1.236 | -3.666 | 0.002 | 0.519 |
| LOC100131298 | 2.067 | 0.009 | 3.413 | 0.003 | 0.519 |
| YY1AP1 | 2.026 | -0.157 | 3.335 | 0.004 | 0.519 |
| KRT18P34 | 3.393 | 4.150 | 3.163 | 0.006 | 0.523 |
| C12orf72 | 2.153 | 1.135 | 3.160 | 0.006 | 0.523 |
| CHIC1 | -2.107 | 0.944 | -3.148 | 0.006 | 0.523 |
| TIMM50 | 2.051 | 1.274 | 3.082 | 0.007 | 0.523 |
| RAP1A | 2.520 | 0.865 | 3.072 | 0.007 | 0.523 |
| AGR2 | -2.134 | 1.589 | -3.047 | 0.007 | 0.523 |
| WDR61 | 2.323 | 1.900 | 3.041 | 0.007 | 0.523 |
| SMPD1 | 2.002 | 0.414 | 3.009 | 0.008 | 0.523 |
| CASQ1 | -2.130 | 0.422 | -2.963 | 0.009 | 0.523 |
| FYTTD1 | 2.157 | 0.122 | 2.951 | 0.009 | 0.523 |
| JSRP1 | -2.203 | 1.604 | -2.889 | 0.010 | 0.523 |
| TCN1 | 2.156 | 0.067 | 2.866 | 0.011 | 0.523 |
| CEP72 | 2.156 | 0.536 | 2.849 | 0.011 | 0.523 |
| MRS2 | -2.187 | 1.760 | -2.822 | 0.012 | 0.523 |
| SNRNP27 | -2.017 | 0.743 | -2.808 | 0.012 | 0.523 |
| GALR2 | -2.273 | 0.475 | -2.762 | 0.013 | 0.528 |
| SPAG4 | -2.371 | 1.115 | -2.742 | 0.014 | 0.532 |
| RBM4 | 2.280 | 1.723 | 2.735 | 0.014 | 0.532 |
| PLA2G1B | -2.290 | 0.946 | -2.731 | 0.014 | 0.532 |
| CEP55 | -2.372 | 0.987 | -2.691 | 0.016 | 0.532 |
| VEZT | -2.505 | 0.062 | -2.669 | 0.016 | 0.534 |
| LOC100130644 | 2.111 | 1.327 | 2.665 | 0.016 | 0.534 |
| NP | 2.016 | 1.971 | 2.663 | 0.016 | 0.534 |
| LOC100133039 | 2.058 | -0.053 | 2.648 | 0.017 | 0.537 |
| ZNF267 | 2.165 | -0.141 | 2.544 | 0.021 | 0.573 |
| LOC441461 | 2.152 | 0.534 | 2.535 | 0.021 | 0.574 |
| GCNT4 | -2.182 | 0.713 | -2.535 | 0.021 | 0.574 |
| FBXO42 | -2.648 | 0.935 | -2.534 | 0.021 | 0.574 |
| RNF126 | 2.042 | 0.481 | 2.474 | 0.024 | 0.582 |
| KRT20 | -2.098 | 0.122 | -2.437 | 0.026 | 0.589 |
| FBXO15 | 2.438 | 0.967 | 2.437 | 0.026 | 0.589 |
| STK40 | 2.238 | 1.339 | 2.326 | 0.033 | 0.614 |
| ERAS | 2.043 | 0.344 | 2.275 | 0.036 | 0.621 |
| MYBPC2 | -2.271 | 0.523 | -2.183 | 0.043 | 0.632 |
| ZWILCH | -2.130 | 0.376 | -2.173 | 0.044 | 0.632 |
| **Obesity** |  |  |  |  |  |
| SNHG12 | 2.087 | 1.705 | 5.916 | 0.000 | 0.082 |
| CTNS | -2.192 | -0.388 | -5.133 | 0.000 | 0.110 |
| JCLN | 2.318 | 1.896 | 4.955 | 0.000 | 0.131 |
| HCG8 | 3.005 | 1.919 | 4.888 | 0.000 | 0.138 |
| CHRNA7 | 2.923 | 1.778 | 4.696 | 0.000 | 0.149 |
| CDR2L | -2.122 | 1.442 | -4.509 | 0.000 | 0.165 |
| LRRC39 | 2.106 | 0.691 | 3.843 | 0.001 | 0.255 |
| ZNF101 | 3.204 | 0.156 | 3.776 | 0.002 | 0.256 |
| SNRPD2 | 2.450 | 2.392 | 3.741 | 0.002 | 0.259 |
| NALCN | 2.371 | 3.420 | 3.677 | 0.002 | 0.264 |
| GRHL2 | 2.483 | 1.385 | 3.639 | 0.002 | 0.272 |
| ZMYND12 | 2.243 | 2.244 | 3.562 | 0.002 | 0.291 |
| JSRP1 | -2.477 | 1.467 | -3.537 | 0.003 | 0.295 |
| DDC | 2.374 | 0.927 | 3.508 | 0.003 | 0.306 |
| LOC100132005 | -2.491 | 0.443 | -3.503 | 0.003 | 0.306 |
| CLDN10 | 2.450 | 1.283 | 3.440 | 0.003 | 0.314 |
| SCAP | -2.194 | 2.822 | -3.437 | 0.003 | 0.315 |
| LOC650392 | 2.268 | 3.262 | 3.405 | 0.003 | 0.320 |
| STK40 | 3.286 | 1.863 | 3.385 | 0.004 | 0.320 |
| PROL1 | -2.228 | 2.892 | -3.352 | 0.004 | 0.325 |
| NFXL1 | -2.079 | 1.153 | -3.302 | 0.004 | 0.337 |
| LOC100128840 | 2.307 | 1.131 | 3.236 | 0.005 | 0.353 |
| LOC100132338 | 2.375 | 0.938 | 3.225 | 0.005 | 0.353 |
| CCDC142 | 2.237 | -0.081 | 3.153 | 0.006 | 0.353 |
| C10orf136 | 2.991 | 0.937 | 3.147 | 0.006 | 0.354 |
| SCGB2A1 | 2.078 | 0.862 | 3.122 | 0.006 | 0.357 |
| MMP26 | 2.146 | 1.532 | 3.112 | 0.006 | 0.360 |
| ZNF384 | -2.031 | -0.003 | -3.099 | 0.007 | 0.362 |
| ZFP57 | -2.371 | 0.762 | -3.074 | 0.007 | 0.363 |
| IL10RB | 2.147 | 1.091 | 3.063 | 0.007 | 0.363 |
| SLC9A7 | 2.227 | 1.027 | 3.008 | 0.008 | 0.364 |
| CD79A | -2.589 | -0.152 | -2.926 | 0.010 | 0.374 |
| LARP6 | -2.094 | 0.734 | -2.924 | 0.010 | 0.374 |
| FAM132A | 2.081 | 1.229 | 2.900 | 0.010 | 0.378 |
| BNIP2 | 2.171 | 1.115 | 2.874 | 0.011 | 0.383 |
| CCDC146 | -2.280 | 0.073 | -2.844 | 0.011 | 0.393 |
| TEKT2 | 2.127 | 4.024 | 2.833 | 0.012 | 0.395 |
| SNRNP27 | -2.119 | 0.692 | -2.755 | 0.014 | 0.405 |
| LRRC29 | -2.382 | 0.124 | -2.748 | 0.014 | 0.405 |
| STRA8 | -2.271 | 0.228 | -2.747 | 0.014 | 0.405 |
| FANCE | -2.211 | 0.958 | -2.721 | 0.015 | 0.405 |
| ALKBH1 | -2.101 | -0.291 | -2.720 | 0.015 | 0.405 |
| MAGEB2 | 2.030 | 1.022 | 2.710 | 0.015 | 0.408 |
| MAK16 | -2.173 | 1.402 | -2.664 | 0.017 | 0.421 |
| ZNF595 | -2.083 | -0.120 | -2.651 | 0.017 | 0.421 |
| TSPYL5 | 2.071 | 2.962 | 2.649 | 0.017 | 0.421 |
| OR6A2 | 2.118 | 1.452 | 2.611 | 0.018 | 0.424 |
| ST6GALNAC5 | 2.102 | 1.584 | 2.524 | 0.022 | 0.456 |
| GAGE1 | 2.272 | 0.222 | 2.461 | 0.025 | 0.472 |
| MRS2 | -2.037 | 1.834 | -2.298 | 0.035 | 0.497 |
| C13orf29 | 2.179 | 0.724 | 2.292 | 0.035 | 0.497 |
| RAP2A | 2.319 | 0.726 | 2.273 | 0.037 | 0.500 |
| TCL1B | 2.062 | 2.376 | 2.195 | 0.043 | 0.518 |
| ENO2 | 2.033 | 0.809 | 2.182 | 0.044 | 0.523 |
| **Obesity with MS** |  |  |  |  |  |
| PTPRH | 3.675 | 1.390 | 13.677 | 0.000 | 0.000 |
| HCG8 | 4.876 | 2.854 | 10.598 | 0.000 | 0.000 |
| CHRNA7 | 4.827 | 2.730 | 10.161 | 0.000 | 0.000 |
| NAPEPLD | 2.443 | 1.040 | 9.294 | 0.000 | 0.000 |
| NDUFA12 | -2.493 | 1.895 | -9.185 | 0.000 | 0.000 |
| LOC100130998 | 3.080 | 2.491 | 9.152 | 0.000 | 0.000 |
| SLC22A17 | 2.082 | 1.049 | 8.161 | 0.000 | 0.001 |
| LOC348021 | 3.076 | 2.160 | 7.769 | 0.000 | 0.001 |
| KCNJ8 | 3.520 | 1.982 | 7.492 | 0.000 | 0.001 |
| C10orf104 | -2.187 | 1.379 | -7.444 | 0.000 | 0.001 |
| TMEM161B | 2.052 | 1.386 | 7.315 | 0.000 | 0.001 |
| GPR17 | -2.259 | 2.389 | -7.137 | 0.000 | 0.001 |
| FGF12 | 2.160 | 1.806 | 6.934 | 0.000 | 0.002 |
| SLC12A9 | 2.111 | 2.353 | 6.608 | 0.000 | 0.003 |
| LOC338809 | 2.670 | 1.626 | 6.508 | 0.000 | 0.003 |
| MKRN2 | 2.191 | 2.194 | 6.403 | 0.000 | 0.003 |
| JCLN | 3.004 | 2.239 | 6.199 | 0.000 | 0.005 |
| CGB2 | 2.251 | 1.795 | 6.067 | 0.000 | 0.005 |
| HOXA11AS | -2.024 | 1.914 | -5.955 | 0.000 | 0.006 |
| STK40 | 5.295 | 2.867 | 5.913 | 0.000 | 0.007 |
| CACNA1E | -2.023 | 0.547 | -5.858 | 0.000 | 0.007 |
| C14orf135 | 4.247 | 2.048 | 5.846 | 0.000 | 0.007 |
| GATA3 | -2.484 | 3.535 | -5.814 | 0.000 | 0.007 |
| RAD9B | -2.686 | 0.633 | -5.747 | 0.000 | 0.008 |
| TRIP12 | 2.045 | 0.958 | 5.663 | 0.000 | 0.009 |
| FOXS1 | -2.029 | 2.579 | -5.609 | 0.000 | 0.009 |
| SNHG1 | 2.231 | 2.851 | 5.399 | 0.000 | 0.011 |
| C2orf56 | 2.988 | 0.757 | 5.284 | 0.000 | 0.014 |
| NDP | -2.094 | 0.603 | -5.193 | 0.000 | 0.016 |
| FBXL14 | 2.113 | 1.229 | 5.058 | 0.000 | 0.019 |
| PDCL | 2.156 | 0.907 | 4.737 | 0.000 | 0.027 |
| AMZ1 | 2.352 | 1.316 | 4.691 | 0.000 | 0.029 |
| CETP | 2.437 | 2.135 | 4.621 | 0.000 | 0.032 |
| ANKRD1 | 2.301 | 0.054 | 4.414 | 0.000 | 0.039 |
| IGLL1 | 2.163 | 0.780 | 4.380 | 0.000 | 0.041 |
| OR10P1 | 2.314 | 2.304 | 4.356 | 0.000 | 0.042 |
| SNHG12 | 2.463 | 1.894 | 4.269 | 0.001 | 0.047 |
| HIST1H3C | -2.035 | 2.109 | -4.249 | 0.001 | 0.048 |
| ITGA6 | 2.722 | 1.030 | 4.019 | 0.001 | 0.062 |
| CALCOCO1 | -2.434 | 2.667 | -3.937 | 0.001 | 0.068 |
| SLC35C1 | 2.259 | 1.107 | 3.899 | 0.001 | 0.071 |
| CFHR1 | 2.301 | 1.701 | 3.898 | 0.001 | 0.071 |
| TMC3 | 2.316 | 1.625 | 3.870 | 0.001 | 0.072 |
| ACSL4 | -2.031 | 1.528 | -3.869 | 0.001 | 0.072 |
| LIMS2 | -2.146 | 1.407 | -3.863 | 0.001 | 0.073 |
| SPATA7 | 2.261 | 0.936 | 3.862 | 0.001 | 0.073 |
| CCDC21 | -2.005 | -0.094 | -3.806 | 0.001 | 0.078 |
| SCFD1 | -2.283 | 0.269 | -3.773 | 0.002 | 0.082 |
| C13orf3 | 2.439 | 3.228 | 3.758 | 0.002 | 0.083 |
| FLJ12616 | -2.477 | 0.731 | -3.739 | 0.002 | 0.085 |
| C18orf51 | -3.265 | 3.027 | -3.731 | 0.002 | 0.085 |
| SOCS5 | -2.298 | 1.732 | -3.696 | 0.002 | 0.088 |
| PIWIL2 | -2.240 | 1.038 | -3.688 | 0.002 | 0.089 |
| HMGCS2 | 2.243 | 1.234 | 3.604 | 0.002 | 0.096 |
| C20orf186 | 3.957 | 2.370 | 3.546 | 0.003 | 0.105 |
| FOXJ3 | -2.241 | 0.840 | -3.423 | 0.003 | 0.122 |
| CAPG | 2.947 | 1.944 | 3.422 | 0.003 | 0.122 |
| GPHN | -2.102 | 1.912 | -3.369 | 0.004 | 0.128 |
| AGR2 | -2.213 | 1.549 | -3.366 | 0.004 | 0.128 |
| FBXO15 | 2.454 | 0.975 | 3.341 | 0.004 | 0.132 |
| LOC100132005 | -2.803 | 0.287 | -3.319 | 0.004 | 0.135 |
| KRT18P34 | 2.900 | 3.904 | 3.279 | 0.005 | 0.140 |
| C10orf136 | 3.032 | 0.958 | 3.256 | 0.005 | 0.142 |
| NP | 2.498 | 2.212 | 3.082 | 0.007 | 0.174 |
| CDR2L | -2.017 | 1.495 | -2.983 | 0.008 | 0.191 |
| 8-Mar | -2.910 | 3.075 | -2.967 | 0.009 | 0.193 |
| JSRP1 | -2.065 | 1.673 | -2.967 | 0.009 | 0.193 |
| FLJ32252 | 2.685 | 1.301 | 2.917 | 0.010 | 0.201 |
| ARGLU1 | -2.851 | 1.149 | -2.912 | 0.010 | 0.202 |
| CA10 | -2.197 | 0.722 | -2.901 | 0.010 | 0.204 |
| KCNQ4 | 2.062 | 0.685 | 2.893 | 0.010 | 0.205 |
| STOML3 | 2.536 | 1.440 | 2.869 | 0.011 | 0.210 |
| FRG2 | 2.371 | 0.879 | 2.853 | 0.011 | 0.213 |
| ZNF101 | 2.499 | -0.196 | 2.838 | 0.012 | 0.215 |
| NSUN5 | -3.241 | 3.720 | -2.819 | 0.012 | 0.218 |
| ATP1A2 | -2.048 | -0.460 | -2.717 | 0.015 | 0.234 |
| LOC728875 | 2.155 | 0.979 | 2.690 | 0.016 | 0.239 |
| RAP1A | 2.076 | 0.643 | 2.679 | 0.016 | 0.240 |
| LOC389332 | 2.589 | 0.809 | 2.647 | 0.017 | 0.248 |
| LASS3 | 2.104 | 0.584 | 2.623 | 0.018 | 0.255 |
| LOC414300 | 2.429 | 1.525 | 2.613 | 0.018 | 0.256 |
| FAP | 2.716 | 1.523 | 2.610 | 0.019 | 0.258 |
| SOX6 | 2.127 | 0.694 | 2.604 | 0.019 | 0.259 |
| SUSD1 | -2.146 | 0.771 | -2.598 | 0.019 | 0.261 |
| DDT | -2.096 | 1.135 | -2.572 | 0.020 | 0.268 |
| KRT6B | -2.283 | 1.540 | -2.564 | 0.020 | 0.269 |
| MUCL1 | 2.071 | 1.812 | 2.562 | 0.020 | 0.269 |
| KIAA0100 | -2.353 | 0.710 | -2.531 | 0.022 | 0.273 |
| C19orf34 | 2.344 | 1.394 | 2.529 | 0.022 | 0.274 |
| ZNF576 | -2.005 | 0.324 | -2.514 | 0.023 | 0.278 |
| ZNF624 | 2.391 | 1.578 | 2.464 | 0.025 | 0.287 |
| MYBPC2 | -2.001 | 0.658 | -2.459 | 0.025 | 0.287 |
| TAAR6 | 2.191 | 0.891 | 2.434 | 0.027 | 0.292 |
| OR2H2 | 2.994 | 0.636 | 2.423 | 0.027 | 0.296 |
| KRTAP12-1 | 2.126 | 1.348 | 2.414 | 0.028 | 0.297 |
| NT5C2 | -2.004 | 1.313 | -2.413 | 0.028 | 0.297 |
| TFAP2B | -2.006 | -0.186 | -2.383 | 0.029 | 0.303 |
| TTBK1 | 2.010 | 0.740 | 2.338 | 0.032 | 0.312 |
| SLC2A6 | -2.023 | 1.312 | -2.335 | 0.032 | 0.313 |
| CEP55 | -2.527 | 0.910 | -2.332 | 0.033 | 0.313 |
| MRS2 | -2.648 | 1.529 | -2.320 | 0.033 | 0.317 |
| HLA-DRA | -2.070 | 0.362 | -2.294 | 0.035 | 0.322 |
| LYPD5 | 2.227 | 1.193 | 2.234 | 0.040 | 0.337 |
| SFXN2 | 2.959 | 1.865 | 2.208 | 0.042 | 0.344 |
| LOC440330 | -2.337 | 0.092 | -2.179 | 0.044 | 0.350 |
| FLJ31715 | 2.249 | 0.426 | 2.163 | 0.045 | 0.354 |
| SPATA22 | 2.092 | 1.419 | 2.163 | 0.045 | 0.354 |
| **Abbreviation:** SAT: subcutaneous adipose tissue; FC: fold change; AveExpr: average of express level; adj.P.Val: adjusted P-value; MS: metabolic syndrome | | | | | |

| **Supplemental table3** Differentially expressed miRNA of overweight, obesity, and obesity with metabolic syndrome in VAT and SAT | | | | | |
| --- | --- | --- | --- | --- | --- |
| **miRNA** | **logFC** | **AveExpr** | **T-value** | **P-value** | **adj.P.Val** |
| **VAT** |  |  |  |  |  |
| hsa-mir-3681 | -0.477 | 2.800 | -3.952 | 0.002 | 0.723 |
| hsa-mir-378h | 0.687 | 1.945 | 3.614 | 0.003 | 0.723 |
| hsa-mir-4517 | -0.580 | 2.572 | -3.493 | 0.004 | 0.723 |
| hsa-mir-758 | -0.355 | 3.134 | -3.461 | 0.005 | 0.723 |
| hsa-mir-3198-2 | -0.662 | 2.545 | -3.392 | 0.005 | 0.723 |
| hsa-mir-3130-2 | -0.398 | 2.339 | -3.294 | 0.006 | 0.723 |
| hsa-mir-597 | -0.429 | 1.622 | -3.240 | 0.007 | 0.723 |
| hsa-mir-3116-2 | -0.579 | 4.526 | -3.190 | 0.008 | 0.723 |
| hsa-mir-185 | 0.263 | 2.544 | 3.172 | 0.008 | 0.723 |
| hsa-mir-4787 | 0.406 | 3.171 | 3.150 | 0.008 | 0.723 |
| hsa-mir-3161 | -0.759 | 2.061 | -3.103 | 0.009 | 0.723 |
| hsa-mir-2681 | -0.394 | 2.223 | -3.062 | 0.010 | 0.723 |
| hsa-mir-5095 | -0.792 | 3.475 | -3.050 | 0.010 | 0.723 |
| hsa-mir-4319 | -0.279 | 4.414 | -3.045 | 0.010 | 0.723 |
| hsa-mir-550b-2 | -0.562 | 5.197 | -2.997 | 0.011 | 0.723 |
| hsa-mir-182 | -0.400 | 3.008 | -2.974 | 0.011 | 0.723 |
| hsa-mir-548an | 0.554 | 1.800 | 2.966 | 0.012 | 0.723 |
| hsa-mir-4487 | 0.375 | 1.951 | 2.902 | 0.013 | 0.723 |
| hsa-mir-654 | -0.353 | 2.803 | -2.855 | 0.014 | 0.723 |
| hsa-mir-550b-1 | -0.510 | 5.648 | -2.788 | 0.016 | 0.723 |
| hsa-mir-568 | -0.463 | 3.999 | -2.781 | 0.016 | 0.723 |
| hsa-mir-4324 | -0.547 | 2.773 | -2.678 | 0.020 | 0.723 |
| hsa-mir-140 | -0.438 | 2.614 | -2.666 | 0.020 | 0.723 |
| hsa-mir-148b | -0.466 | 1.572 | -2.653 | 0.021 | 0.723 |
| hsa-mir-3620 | -0.670 | 3.548 | -2.641 | 0.021 | 0.723 |
| hsa-mir-32 | 0.472 | 3.316 | 2.632 | 0.022 | 0.723 |
| hsa-mir-4432 | -0.268 | 1.852 | -2.600 | 0.023 | 0.723 |
| hsa-mir-4269 | -0.701 | 2.537 | -2.594 | 0.023 | 0.723 |
| hsa-mir-4669 | 0.290 | 2.299 | 2.579 | 0.024 | 0.723 |
| hsa-mir-4723 | -0.375 | 1.871 | -2.578 | 0.024 | 0.723 |
| hsa-mir-3913-2 | 0.288 | 1.527 | 2.568 | 0.024 | 0.723 |
| hsa-mir-532 | -0.291 | 3.247 | -2.566 | 0.024 | 0.723 |
| hsa-mir-16-2 | 0.329 | 3.229 | 2.566 | 0.024 | 0.723 |
| hsa-mir-148a | -0.479 | 1.874 | -2.513 | 0.027 | 0.749 |
| hsa-mir-218-2 | -0.366 | 1.439 | -2.503 | 0.027 | 0.749 |
| hsa-mir-656 | -0.310 | 3.828 | -2.482 | 0.029 | 0.749 |
| hsa-mir-4742 | 0.355 | 2.738 | 2.448 | 0.030 | 0.749 |
| hsa-mir-4707 | 0.452 | 1.866 | 2.448 | 0.030 | 0.749 |
| hsa-mir-138-2 | -0.554 | 2.261 | -2.424 | 0.032 | 0.749 |
| hsa-mir-4305 | -0.518 | 2.473 | -2.416 | 0.032 | 0.749 |
| hsa-mir-3202-2 | -0.608 | 3.418 | -2.411 | 0.033 | 0.749 |
| hsa-mir-4252 | -0.688 | 2.272 | -2.410 | 0.033 | 0.749 |
| hsa-mir-4667 | -0.594 | 2.714 | -2.408 | 0.033 | 0.749 |
| hsa-mir-4635 | -0.836 | 3.761 | -2.394 | 0.034 | 0.757 |
| hsa-mir-4800 | 0.345 | 2.862 | 2.366 | 0.035 | 0.760 |
| hsa-mir-4429 | 0.445 | 1.808 | 2.358 | 0.036 | 0.760 |
| hsa-mir-3922 | -0.337 | 2.108 | -2.324 | 0.038 | 0.769 |
| hsa-mir-4782 | -0.560 | 2.410 | -2.304 | 0.040 | 0.769 |
| hsa-mir-3613 | 0.371 | 6.978 | 2.288 | 0.041 | 0.769 |
| hsa-mir-566 | -0.931 | 5.788 | -2.254 | 0.043 | 0.769 |
| hsa-mir-4785 | 0.293 | 1.593 | 2.217 | 0.046 | 0.801 |
| hsa-mir-3938 | -0.431 | 1.746 | -2.205 | 0.047 | 0.810 |
| **SAT** |  |  |  |  |  |
| hsa-miR-1308_st | -0.935 | 8.673 | -4.345 | 0.000 | 0.042 |
| hsa-miR-886-5p_st | -0.772 | 5.921 | -4.185 | 0.000 | 0.042 |
| hsa-miR-222_st | -0.444 | 7.858 | -3.531 | 0.001 | 0.147 |
| hsa-let-7d_st | 0.368 | 9.663 | 3.522 | 0.001 | 0.147 |
| hsa-miR-342-3p_st | -0.276 | 8.818 | -3.493 | 0.001 | 0.147 |
| hsa-miR-143_st | 0.540 | 7.768 | 3.456 | 0.001 | 0.147 |
| hsa-miR-196a_st | 0.356 | 4.381 | 3.010 | 0.004 | 0.422 |
| hsa-miR-26a_st | 0.371 | 8.860 | 2.884 | 0.006 | 0.520 |
| hsa-miR-16_st | 0.762 | 8.323 | 2.835 | 0.006 | 0.535 |
| hsa-miR-378_st | 0.298 | 8.651 | 2.727 | 0.008 | 0.617 |
| hsa-miR-652_st | 0.476 | 7.535 | 2.715 | 0.009 | 0.617 |
| hsa-miR-422a_st | 0.717 | 6.140 | 2.577 | 0.013 | 0.637 |
| hsa-miR-485-3p_st | 0.377 | 4.896 | 2.570 | 0.013 | 0.637 |
| hsa-miR-140-3p_st | -0.364 | 8.132 | -2.506 | 0.015 | 0.671 |
| hsa-miR-152_st | 0.382 | 4.724 | 2.323 | 0.024 | 0.719 |
| hsa-miR-10b_st | 0.275 | 4.664 | 2.323 | 0.024 | 0.719 |
| hsa-miR-451_st | 0.647 | 5.365 | 2.307 | 0.025 | 0.719 |
| hsa-miR-483-5p_st | -0.421 | 5.564 | -2.244 | 0.029 | 0.719 |
| hsa-let-7g_st | 0.624 | 5.794 | 2.231 | 0.030 | 0.719 |
| hsa-miR-126_st | 0.533 | 7.446 | 2.168 | 0.034 | 0.719 |
| hsa-miR-877-star_st | -0.279 | 4.519 | -2.144 | 0.036 | 0.719 |
| hsa-miR-634_st | -0.293 | 4.642 | -2.123 | 0.038 | 0.719 |
| hsa-miR-425_st | 0.431 | 6.020 | 2.121 | 0.038 | 0.719 |
| hsa-let-7i_st | 0.573 | 7.191 | 2.058 | 0.044 | 0.719 |
| hsa-miR-595_st | 0.340 | 4.775 | 2.037 | 0.046 | 0.719 |
| **Abbreviation:** VAT: visceral adipose tissue; SAT: subcutaneous adipose tissue; FC: fold change; AveExpr: average of express level; adj.P.Val: adjusted P-value | | | | | |

| **Supplemental table4** Differentially expressed mRNAs of ccRCC among target mRNAs in networks of obesity in VAT and SAT | | | | | |
| --- | --- | --- | --- | --- | --- |
| **mRNA** | **logFC** | **AveExpr** | **T-value** | **P-value** | **adj.P.Val** |
| **VAT** |  |  |  |  |  |
| SH3GL2 | -2.270 | 5.379 | -6.512 | 0.000 | 0.000 |
| ASS1 | -2.702 | 10.733 | -5.642 | 0.000 | 0.000 |
| CALCRL | 1.449 | 5.998 | 5.529 | 0.000 | 0.000 |
| GBP2 | 1.814 | 6.111 | 5.200 | 0.000 | 0.001 |
| ARPC5 | 0.501 | 8.054 | 5.093 | 0.000 | 0.001 |
| GMPR | -1.339 | 6.035 | -4.941 | 0.000 | 0.001 |
| MFSD3 | -0.770 | 5.569 | -4.720 | 0.000 | 0.001 |
| CTSO | 0.626 | 9.003 | 3.916 | 0.001 | 0.006 |
| HIBADH | -0.674 | 7.446 | -3.724 | 0.001 | 0.008 |
| RAB33A | 0.394 | 4.547 | 3.397 | 0.002 | 0.015 |
| PSMA5 | -0.290 | 6.850 | -3.395 | 0.002 | 0.015 |
| ISCU | -0.657 | 10.866 | -3.194 | 0.004 | 0.022 |
| MTTP | -0.960 | 4.200 | -3.186 | 0.004 | 0.022 |
| PCDHAC1 | 0.167 | 4.448 | 3.184 | 0.004 | 0.022 |
| RAD54L2 | -0.194 | 7.070 | -3.044 | 0.006 | 0.029 |
| ATP2B2 | 0.243 | 4.612 | 2.942 | 0.007 | 0.034 |
| FLRT3 | -0.870 | 7.982 | -2.884 | 0.008 | 0.038 |
| PDHX | -0.431 | 7.778 | -2.792 | 0.010 | 0.045 |
| FNIP1 | 0.206 | 4.717 | 2.685 | 0.013 | 0.054 |
| MAP3K8 | 0.617 | 4.853 | 2.629 | 0.015 | 0.059 |
| ZAN | 0.201 | 5.015 | 2.558 | 0.017 | 0.067 |
| SORCS1 | -0.466 | 4.602 | -2.487 | 0.020 | 0.076 |
| CDH2 | 0.643 | 7.515 | 2.435 | 0.023 | 0.082 |
| CD160 | 0.157 | 4.221 | 2.423 | 0.023 | 0.084 |
| PRPSAP1 | -0.179 | 6.794 | -2.372 | 0.026 | 0.091 |
| GAGE1 | -0.104 | 3.682 | -2.267 | 0.033 | 0.108 |
| CATSPER1 | 0.160 | 5.202 | 2.126 | 0.044 | 0.134 |
| MMP25 | -0.146 | 6.199 | -2.102 | 0.046 | 0.140 |
| **SAT** |  |  |  |  |  |
| IL10RB | 1.066 | 6.703 | 6.625 | 0.000 | 0.000 |
| TFAP2B | -1.597 | 4.772 | -6.265 | 0.000 | 0.000 |
| ENO2 | 2.722 | 6.833 | 6.065 | 0.000 | 0.000 |
| SCAP | -0.728 | 8.100 | -6.015 | 0.000 | 0.000 |
| GATA3 | -2.137 | 6.185 | -5.540 | 0.000 | 0.000 |
| DMRT2 | -1.845 | 5.370 | -5.187 | 0.000 | 0.001 |
| CA10 | -1.000 | 4.386 | -5.121 | 0.000 | 0.001 |
| CLDN10 | -1.745 | 8.855 | -4.976 | 0.000 | 0.001 |
| FOXJ3 | 1.302 | 6.825 | 4.837 | 0.000 | 0.001 |
| SFXN2 | -1.153 | 6.375 | -4.788 | 0.000 | 0.001 |
| SLC22A17 | -0.309 | 5.614 | -4.706 | 0.000 | 0.001 |
| CTNS | -0.339 | 5.892 | -4.556 | 0.000 | 0.002 |
| ZNF267 | 0.993 | 5.793 | 4.526 | 0.000 | 0.002 |
| PDCL | 0.348 | 5.653 | 4.448 | 0.000 | 0.002 |
| ACSL4 | -0.506 | 5.041 | -4.221 | 0.000 | 0.003 |
| NAPEPLD | -0.635 | 5.603 | -4.158 | 0.000 | 0.004 |
| GPHN | -0.836 | 6.819 | -4.156 | 0.000 | 0.004 |
| RBM4 | 0.592 | 5.863 | 3.937 | 0.001 | 0.006 |
| RAP2A | 0.325 | 6.103 | 3.843 | 0.001 | 0.007 |
| ITGA6 | 0.566 | 8.433 | 3.688 | 0.001 | 0.009 |
| GRHL2 | -0.712 | 5.519 | -3.621 | 0.001 | 0.010 |
| VEZT | 0.351 | 5.593 | 3.270 | 0.003 | 0.019 |
| TTBK1 | -0.182 | 6.025 | -3.263 | 0.003 | 0.019 |
| TSPYL5 | -1.238 | 7.033 | -3.255 | 0.003 | 0.020 |
| DDC | -1.828 | 7.735 | -3.252 | 0.003 | 0.020 |
| BNIP2 | 0.534 | 6.349 | 3.231 | 0.004 | 0.020 |
| CEP55 | 0.570 | 3.617 | 3.162 | 0.004 | 0.023 |
| SMPD1 | -0.415 | 6.416 | -3.133 | 0.005 | 0.024 |
| SOX6 | -0.332 | 5.582 | -3.087 | 0.005 | 0.026 |
| FYTTD1 | 0.344 | 7.978 | 3.078 | 0.005 | 0.027 |
| NT5C2 | -0.653 | 8.001 | -3.076 | 0.005 | 0.027 |
| HMGCS2 | -1.347 | 6.993 | -3.054 | 0.005 | 0.028 |
| ALKBH1 | 0.163 | 5.512 | 3.032 | 0.006 | 0.029 |
| SNRNP27 | 0.448 | 8.222 | 2.771 | 0.011 | 0.046 |
| SUSD1 | -0.395 | 6.674 | -2.558 | 0.017 | 0.067 |
| SOCS5 | 0.394 | 6.394 | 2.521 | 0.019 | 0.071 |
| FBXL14 | 0.177 | 5.022 | 2.505 | 0.019 | 0.073 |
| LYPD5 | 0.190 | 4.390 | 2.279 | 0.032 | 0.106 |
| GAGE1 | -0.104 | 3.682 | -2.267 | 0.033 | 0.108 |
| RAP1A | 0.140 | 6.414 | 2.229 | 0.035 | 0.114 |
| ZNF576 | 0.097 | 6.244 | 2.143 | 0.042 | 0.131 |
| **Abbreviation:** ccRCC: clear cell renal cell carcinoma; VAT: visceral adipose tissue; SAT: subcutaneous adipose tissue; FC: fold change; AveExpr: average of express level; adj.P.Val: adjusted P-value | | | | | |

| **Supplemental table5** Verification of RNAs in miRNA-mRNA networks of obesity-related ccRCC in VAT and SAT using TCGA dataset | | | | | |
| --- | --- | --- | --- | --- | --- |
| **RNA** | **AUC** | **95%CI of AUC** | **OR** | **95%CI of OR** | **P value** |
| **mRNA of VAT** |  |  |  |  |  |
| PRPSAP1 | 0.903 | 0.869, 0.937 | 0.407 | 0.337, 0.492 | <0.001 |
| SORCS1 | 0.785 | 0.732, 0.838 | 0.22 | 0.136, 0.354 | <0.001 |
| FLRT3 | 0.87 | 0.836, 0.903 | 0.937 | 0.914, 0.962 | <0.001 |
| MTTP | 0.886 | 0.859, 0.913 | 0.837 | 0.765, 0.916 | <0.001 |
| ISCU | 0.918 | 0.891, 0.945 | 0.946 | 0.930, 0.963 | <0.001 |
| HIBADH | 0.931 | 0.897, 0.966 | 0.934 | 0.922, 0.947 | <0.001 |
| CATSPER1 | 0.858 | 0.813, 0.904 | N/A | N/A | <0.001 |
| CD160 | 0.916 | 0.880, 0.952 | N/A | N/A | <0.001 |
| CDH2 | 0.869 | 0.836, 0.901 | 1.178 | 1.134, 1.224 | <0.001 |
| ZAN | 0.751 | 0.691, 0.810 | N/A | N/A | 0.001 |
| MAP3K8 | 0.765 | 0.711, 0.819 | 2.681 | 1.926, 3.370 | <0.001 |
| ATP2B2 | 0.861 | 0.828, 0.895 | 45.21 | 13.26, 154.13 | <0.001 |
| RAB33A | 0.881 | 0.842, 0.921 | 47.21 | 17.79, 125.31 | <0.001 |
| **miRNA of VAT** |  |  |  |  |  |
| hsa-mir-32 | 0.826 | 0.781, 0.870 | 4.96 | 3.24, 7.59 | <0.001 |
| hsa-mir-185 | 0.875 | 0.834, 0.916 | 16.28 | 8.92, 29.72 | <0.001 |
| hsa-mir-532 | 0.887 | 0.850, 0.924 | 0.16 | 0.109, 0.235 | <0.001 |
| hsa-mir-758 | 0.672 | 0.618, 0.725 | 0.627 | 0.502, 0.783 | <0.001 |
| hsa-mir-656 | 0.598 | 0.533, 0.662 | 0.553 | 0.387, 0.790 | 0.001 |
| hsa-mir-654 | 0.575 | 0.514, 0.637 | 0.784 | 0.644, 0.955 | 0.015 |
| hsa-mir-3938 | 0.539 | 0.506, 0.571 | 0.094 | 0.019, 0.461 | 0.004 |
| hsa-mir-182 | 0.63 | 0.577, 0.583 | 0.808 | 0.694, 0.941 | 0.006 |
| **mRNA of SAT** |  |  |  |  |  |
| LYPD5 | 0.589 | 0.527, 0.651 | 4 | 1.40, 11.45 | 0.01 |
| BNIP2 | 0.667 | 0.610, 0.723 | 1.25 | 1.10, 1.41 | <0.001 |
| ITGA6 | 0.615 | 0.567, 0.662 | 1.03 | 1.01, 1.05 | <0.001 |
| RAP2A | 0.773 | 0.724, 0.821 | 1.31 | 1.21, 1.41 | <0.001 |
| PDCL | 0.601 | 0.548, 0.654 | 1.17 | 1.05, 1.32 | 0.007 |
| ZNF267 | 0.901 | 0.862, 0.940 | 11.56 | 6.60, 20.25 | <0.001 |
| ENO2 | 0.963 | 0.945, 0.981 | 1.28 | 1.20, 1.36 | <0.001 |
| IL10RB | 0.949 | 0.925, 0.972 | 1.64 | 1.48, 1.82 | <0.001 |
| SUSD1 | 0.938 | 0.917, 0.959 | 0.748 | 0.702, 0.798 | <0.001 |
| NT5C2 | 0.944 | 0.921, 0.967 | 0.341 | 0.276, 0.422 | <0.001 |
| GPHN | 0.902 | 0.870, 0.934 | 0.864 | 0.810, 0.921 | <0.001 |
| ACSL4 | 0.968 | 0.954, 0.982 | 0.84 | 0.812, 0.869 | <0.001 |
| CTNS | 0.837 | 0.790, 0.883 | 0.548 | 0.475, 0.633 | <0.001 |
| CA10 | 0.986 | 0.975, 0.996 | 0.251 | 0.191, 0.331 | <0.001 |
| GATA3 | 0.975 | 0.958, 0.991 | 0.833 | 0.805, 0.862 | <0.001 |
| SCAP | 0.945 | 0.922, 0.967 | 0.603 | 0.548, 0.664 | <0.001 |
| TFAP2B | 0.991 | 0.979, 1.000 | 0.185 | 0.125, 0.274 | <0.001 |
| **miRNA of SAT** |  |  |  |  |  |
| hsa-mir-425 | 0.598 | 0.523, 0.674 | 1.47 | 1.09, 1.99 | 0.013 |
| hsa-mir-126 | 0.674 | 0.626, 0.723 | 1.51 | 1.17, 1.96 | 0.002 |
| miRNA and mRNA shown in the table have statistically significant ORs, and same trends (up-or down-regulated) as shown in the networks. **Abbreviation:** ccRCC: clear cell renal cell carcinoma; VAT: visceral adipose tissue; SAT: subcutaneous adipose tissue; AUC: area under curve of ROC; OR: odd ratio; 95%CI: 95% confidence interval | | | | | |

| **Supplemental table6** Associations of differentially expressed RNAs of obesity-related ccRCC in VAT and SAT with survival of ccRCC | | | | | | |
| --- | --- | --- | --- | --- | --- | --- |
| **RNA** | **Crude HR** | **95%CI of crude HR** | **P value** | **Adjusted HR** | **95%CI of adjusted HR** | **P value** |
| **mRNA of VAT** |  |  |  |  |  |  |
| ADA | 1.02 | 1.01, 1.03 | 0.008 | 1.01 | 1.00, 1.02 | 0.21 |
| ATP2B2 | 0.93 | 0.849, 1.018 | 0.114 | 0.963 | 0.879, 1.056 | 0.423 |
| CATSPER1 | 2.19 | 1.46, 3.29 | <0.001 | 2.3 | 1.48, 3.58 | <0.001 |
| CD160 | 0.818 | 0.433, 1.545 | 0.536 | 0.842 | 0.418, 1.696 | 0.631 |
| CDH2 | 0.991 | 0.976, 1.006 | 0.253 | 0.988 | 0.972, 1.003 | 0.117 |
| MAP3K8 | 1.18 | 1.12, 1.25 | <0.001 | 1.14 | 1.08, 1.21 | <0.001 |
| RAB33A | 0.892 | 0.792, 1.004 | 0.057 | 0.83 | 0.725, 0.951 | 0.007 |
| ZAN | 0.852 | 0.365, 1.991 | 0.712 | 0.811 | 0.246, 2.675 | 0.731 |
| CETN2 | 1.02 | 1.00, 1.03 | 0.035 | 1.01 | 0.99, 1.02 | 0.354 |
| FLRT3 | 0.94 | 0.913, 0.968 | <0.001 | 0.947 | 0.921, 0.974 | <0.001 |
| HIBADH | 0.983 | 0.974, 0.992 | <0.001 | 0.984 | 0.974, 0.993 | <0.001 |
| ISCU | 1 | 0.99, 1.01 | 0.724 | 1.01 | 0.99, 1.02 | 0.45 |
| MMP25 | 1.04 | 0.98, 1.11 | 0.164 | 1.02 | 0.95, 1.08 | 0.645 |
| MTTP | 1.06 | 1.01, 1.10 | 0.008 | 1.04 | 0.99, 1.10 | 0.097 |
| NECAB2 | 1.03 | 0.98, 1.08 | 0.253 | 1.01 | 0.95, 1.07 | 0.737 |
| PRPSAP1 | 1.09 | 0.95, 1.26 | 0.22 | 1.22 | 1.06, 1.41 | 0.006 |
| SORCS1 | 0.479 | 0.237, 0.968 | 0.04 | 0.392 | 0.189, 0.812 | 0.012 |
| **miRNA of VAT** |  |  |  |  |  |  |
| hsa-mir-3681 | 0.85 | 0.587, 1.232 | 0.39 | 0.825 | 0.569, 1.196 | 0.309 |
| hsa-mir-4517 | 1.58 | 1.00, 2.49 | 0.05 | 1.61 | 1.00, 2.58 | 0.049 |
| hsa-mir-758 | 0.984 | 0.873, 1.108 | 0.788 | 1.04 | 0.92, 1.17 | 0.562 |
| hsa-mir-3198-2 | 1.77 | 1.11, 2.83 | 0.017 | 1.57 | 0.98, 2.53 | 0.063 |
| hsa-mir-3130-2 | 0.881 | 0.771, 1.007 | 0.063 | 0.818 | 0.716, 0.936 | 0.004 |
| hsa-mir-597 | 0.98 | 0.706, 1.360 | 0.903 | 0.921 | 0.652, 1.302 | 0.642 |
| hsa-mir-3116-2 | 0.432 | 0.189, 0.987 | 0.046 | 0.523 | 0.228, 1.200 | 0.126 |
| hsa-mir-185 | 0.972 | 0.769, 1.227 | 0.809 | 1.03 | 0.80, 1.32 | 0.84 |
| hsa-mir-4787 | 1.135 | 0.924, 1.393 | 0.227 | 0.953 | 0.772, 1.177 | 0.656 |
| hsa-mir-3161 | 0.825 | 0.656, 1.037 | 0.1 | 0.815 | 0.638, 1.041 | 0.101 |
| hsa-mir-2681 | 0.88 | 0.544, 1.421 | 0.601 | 0.595 | 0.367, 0.964 | 0.035 |
| hsa-mir-5095 | N/A | N/A | 0.98 | N/A | N/A | 0.975 |
| hsa-mir-550b-2 | 0.968 | 0.120, 7.829 | 0.976 | 1.41 | 0.16, 12.05 | 0.757 |
| hsa-mir-182 | 1.19 | 1.09, 1.30 | <0.001 | 1.14 | 1.04, 1.26 | 0.007 |
| hsa-mir-548an | 0.616 | 0.086, 4.420 | 0.63 | 0.917 | 0.123, 6.860 | 0.933 |
| hsa-mir-4487 | 1.7 | 1.01, 2.88 | 0.048 | 1.87 | 1.13, 3.12 | 0.016 |
| hsa-mir-654 | 1.08 | 0.96, 1.22 | 0.19 | 1.13 | 1.00, 1.28 | 0.044 |
| hsa-mir-550b-1 | 0.527 | 0.142, 1.959 | 0.339 | 0.72 | 0.202, 2.565 | 0.612 |
| hsa-mir-568 | N/A | N/A | 0.985 | N/A | N/A | 0.99 |
| hsa-mir-140 | 0.821 | 0.640, 1.054 | 0.122 | 0.949 | 0.734, 1.228 | 0.692 |
| hsa-mir-148b | 0.881 | 0.605, 1.089 | 0.164 | 0.715 | 0.525, 0.975 | 0.034 |
| hsa-mir-3620 | 0.946 | 0.661, 1.355 | 0.764 | 1.24 | 0.85, 1.80 | 0.268 |
| hsa-mir-32 | 0.863 | 0.711, 1.047 | 0.136 | 0.925 | 0.742, 1.153 | 0.489 |
| hsa-mir-4669 | 1.01 | 0.39, 2.64 | 0.988 | 1.54 | 0.53, 4.49 | 0.431 |
| hsa-mir-4723 | 0.377 | 0.143, 0.989 | 0.048 | 0.38 | 0.141, 1.021 | 0.055 |
| hsa-mir-3913-2 | 0.898 | 0.751, 1.073 | 0.237 | 1.01 | 0.84, 1.22 | 0.897 |
| hsa-mir-532 | 0.707 | 0.562, 0.888 | 0.003 | 0.805 | 0.629, 1.031 | 0.086 |
| hsa-mir-16-2 | 1.05 | 0.78, 1.41 | 0.752 | 1.07 | 0.78, 1.46 | 0.666 |
| hsa-mir-148a | 1.26 | 1.06, 1.50 | 0.008 | 0.951 | 0.783, 1.155 | 0.61 |
| hsa-mir-218-2 | 1.05 | 0.94, 1.18 | 0.402 | 1.16 | 1.02, 1.32 | 0.022 |
| hsa-mir-656 | 1.19 | 0.91, 1.54 | 0.201 | 1.15 | 0.88, 1.49 | 0.319 |
| hsa-mir-4742 | 1 | 0.84, 1.19 | 0.988 | 1.03 | 0.87, 1.23 | 0.736 |
| hsa-mir-4707 | 1.02 | 0.96, 1.51 | 0.113 | 1.06 | 0.84, 1.33 | 0.64 |
| hsa-mir-138-2 | 1.23 | 1.08, 1.39 | 0.001 | 1.15 | 1.01, 1.31 | 0.031 |
| hsa-mir-3202-2 | 0.44 | 0.189, 1.024 | 0.057 | 0.497 | 0.217, 1.136 | 0.096 |
| hsa-mir-4667 | 1.35 | 0.77, 2.36 | 0.29 | 1.13 | 0.64, 1.98 | 0.677 |
| hsa-mir-4635 | 0.434 | 0.145, 1.296 | 0.135 | 0.61 | 0.205, 1.815 | 0.375 |
| hsa-mir-4800 | 1.12 | 0.83, 1.52 | 0.468 | 1.01 | 0.752, 1.363 | 0.934 |
| hsa-mir-4429 | N/A | N/A | 0.975 | N/A | N/A | 0.977 |
| hsa-mir-3922 | 1.36 | 1.12, 1.64 | 0.002 | 1.2 | 1.00, 1.46 | 0.057 |
| hsa-mir-4782 | 1.07 | 0.73, 1.58 | 0.714 | 0.97 | 0.65, 1.43 | 0.866 |
| hsa-mir-3613 | 1.49 | 1.15, 1.92 | 0.003 | 1.31 | 1.01, 1.69 | 0.042 |
| hsa-mir-4785 | 0.55 | 0.302, 1.002 | 0.051 | 0.442 | 0.243, 0.803 | 0.007 |
| hsa-mir-3989 | N/A | N/A | 0.687 | N/A | N/A | 0.742 |
| **mRNA of SAT** |  |  |  |  |  |  |
| BNIP2 | 0.977 | 0.919, 1.038 | 0.445 | 1.01 | 0.94, 1.08 | 0.867 |
| ENO2 | 1.01 | 1.00, 1.01 | <0.001 | 1.01 | 1.00, 1.01 | 0.001 |
| FAP | 1.2 | 1.09, 1.33 | <0.001 | 1.12 | 1.01, 1.24 | 0.027 |
| FBXL14 | 0.875 | 0.796, 0.961 | 0.005 | 0.931 | 0.842, 1.031 | 0.169 |
| FYTTD1 | 0.963 | 0.908, 1.022 | 0.216 | 0.977 | 0.921, 1.037 | 0.448 |
| IL10RB | 1.06 | 1.03, 1.09 | <0.001 | 1.04 | 1.01, 1.07 | 0.011 |
| ITGA6 | 0.971 | 0.962, 0.981 | <0.001 | 0.981 | 0.972, 0.991 | <0.001 |
| KCNJ8 | 1.02 | 1.00, 1.04 | 0.103 | 1.02 | 1.00, 1.04 | 0.12 |
| LYPD5 | 0.299 | 0.161, 0.556 | <0.001 | 0.386 | 0.208, 0.714 | 0.002 |
| PDCL | 0.861 | 0.805, 0.922 | <0.001 | 0.923 | 0.857, 0.994 | 0.035 |
| RAP1A | 0.955 | 0.917, 0.995 | 0.026 | 0.94 | 0.898, 0.983 | 0.007 |
| RAP2A | 0.913 | 0.880, 0.948 | <0.001 | 0.925 | 0.886, 0.965 | <0.001 |
| RBM4 | 0.82 | 0.640, 1.050 | 0.115 | 0.939 | 0.736, 1.197 | 0.609 |
| RNF126 | 1.08 | 1.03, 1.14 | 0.002 | 1.04 | 0.98, 1.09 | 0.192 |
| YY1AP1 | 1.07 | 0.99, 1.15 | 0.08 | 1.09 | 1.02, 1.18 | 0.019 |
| ZNF267 | 1.04 | 0.93, 1.16 | 0.529 | 0.95 | 0.834, 1.082 | 0.439 |
| ACSL4 | 1.01 | 0.98, 1.03 | 0.637 | 1.01 | 0.99, 1.03 | 0.579 |
| CA10 | 0.547 | 0.228, 1.312 | 0.177 | 0.726 | 0.351, 1.503 | 0.389 |
| CTNS | 1.08 | 0.99, 1.19 | 0.079 | 1.11 | 1.01, 1.22 | 0.038 |
| GATA3 | 0.997 | 0.979, 1.015 | 0.726 | 1 | 0.99, 1.02 | 0.653 |
| GPHN | 0.996 | 0.961, 1.033 | 0.845 | 1.02 | 1.00, 1.04 | 0.116 |
| NT5C2 | 0.913 | 0.819, 1.018 | 0.101 | 0.986 | 0.886, 1.098 | 0.801 |
| SCAP | 1.03 | 0.98, 1.09 | 0.258 | 1.07 | 1.01, 1.13 | 0.02 |
| TFAP2B | 0.653 | 0.369, 1.155 | 0.143 | 0.803 | 0.470, 1.371 | 0.421 |
| **miRNA of SAT** |  |  |  |  |  |  |
| hsa-mir-222 | 1.19 | 1.05, 1.36 | 0.008 | 1.34 | 1.17, 1.55 | <0.001 |
| hsa-let-7d | 0.93 | 0.67, 1.31 | 0.69 | 1.14 | 0.82, 1.58 | 0.433 |
| hsa-mir-143 | 0.822 | 0.711, 0.950 | 0.008 | 0.857 | 0.741, 0.991 | 0.038 |
| hsa-mir-652 | 0.866 | 0.659, 1.137 | 0.299 | 1 | 0.76, 1.33 | 0.992 |
| hsa-mir-422a | N/A | N/A | 0.982 | N/A | N/A | 0.981 |
| hsa-mir-152 | 1.08 | 0.89， 1.31 | 0.428 | 1.04 | 0.85, 1.27 | 0.678 |
| hsa-mir-10b | 0.593 | 0.487, 0.723 | <0.001 | 0.703 | 0.572, 0.866 | <0.001 |
| hsa-let-7g | 1.09 | 0.85, 1.41 | 0.487 | 1.15 | 0.87, 1.52 | 0.336 |
| hsa-mir-126 | 0.797 | 0.680, 0.935 | 0.005 | 0.881 | 0.743, 1.044 | 0.144 |
| hsa-mir-425 | 1.37 | 1.16, 1.62 | <0.001 | 1.36 | 1.14, 1.62 | <0.001 |
| hsa-mir-7i | 1.73 | 1.27, 2.35 | <0.001 | 1.21 | 0.87, 1.70 | 0.257 |
| miRNA and mRNA shown in the table are differentially expressed RNA of obesity-related ccRCC screened out using TCGA dataset. Crude HR was unadjusted, adjusted HR was adjusted for age, gender, as well as the tumor stage according to the tumor-node-metastasis (TNM) staging system of ccRCC. **Abbreviation:** ccRCC: clear cell renal cell carcinoma; VAT: visceral adipose tissue; SAT: subcutaneous adipose tissue; HR: hazard ratio; 95%CI: 95% confidence interval | | | | | | |
